# Plasmid Metagenome Reveals High Levels of Antibiotic Resistance Genes and Mobile Genetic Elements in Activated Sludge

**DOI:** 10.1371/journal.pone.0026041

**Published:** 2011-10-10

**Authors:** Tong Zhang, Xu-Xiang Zhang, Lin Ye

**Affiliations:** Environmental Biotechnology Lab, Department of Civil Engineering, The University of Hong Kong, Hong Kong SAR, China; Argonne National Laboratory, United States of America

## Abstract

The overuse or misuse of antibiotics has accelerated antibiotic resistance, creating a major challenge for the public health in the world. Sewage treatment plants (STPs) are considered as important reservoirs for antibiotic resistance genes (ARGs) and activated sludge characterized with high microbial density and diversity facilitates ARG horizontal gene transfer (HGT) via mobile genetic elements (MGEs). However, little is known regarding the pool of ARGs and MGEs in sludge microbiome. In this study, the transposon aided capture (TRACA) system was employed to isolate novel plasmids from activated sludge of one STP in Hong Kong, China. We also used Illumina Hiseq 2000 high-throughput sequencing and metagenomics analysis to investigate the plasmid metagenome. Two novel plasmids were acquired from the sludge microbiome by using TRACA system and one novel plasmid was identified through metagenomics analysis. Our results revealed high levels of various ARGs as well as MGEs for HGT, including integrons, transposons and plasmids. The application of the TRACA system to isolate novel plasmids from the environmental metagenome, coupled with subsequent high-throughput sequencing and metagenomic analysis, highlighted the prevalence of ARGs and MGEs in microbial community of STPs.

## Introduction

The wide use of antibiotics results in environmental releases, accelerating antibiotic resistance development in hospital wastewater, domestic sewage and livestock manure. Sewage receives the gut bacteria previously exposed to antibiotics, so that sewage treatment plants (STPs) are considered as important reservoirs for antibiotic resistance genes (ARGs) [1]. Mobile genetic elements (MGEs), e.g. plasmids [2], [3], transposons [3] and integrons [4], [5], are often involved in horizontal transfer of ARGs among environmental bacteria. With the help of insertion sequences (ISs), transposons often jump randomly and occasionally on genome or plasmid, resulting in gene transfer and multiple resistances [6]. Integrons may capture, integrate and express resistance gene cassettes in their variable region and facilitate the transmission of resistance genes via transposons or conjugative plasmids [7], [8]. Integrons carrying multiple ARGs are frequently detected in the environments of STPs [4], [9], [10], [11].

Activated sludge and biofilm in STPs are characterized with high microbial density and diversity which may facilitate ARG horizontal transfer [12]. Previous studies have shown that plasmid diversity is extremely high in the microbial community of STPs [3], [9], [12], [13], [14], [15], [16]. However, these investigations were carried out using culture-dependent methods, thus genomic analysis is only possible for a limited selected subset of plasmids. Furthermore, investigation of antibiotic resistance in microbial communities based solely on cultivable bacteria makes the assessment results unrepresentative and biased [17]. Therefore, a metagenomic approach is needed for a more comprehensive overview of plasmids residing in STP microorganisms. Recent studies have shown that the high-throughput sequencing is a promising tool for the analysis of complex microbial communities [17], [18].

In this study, we employed the transposon aided capture (TRACA) system [19] to isolate novel plasmids from microbial communities in activated sludge of a STP of Hong Kong, China. High-throughput sequencing and metagenomic analyses were also employed to investigate the diversity and relative abundances of ARGs, virulent factors (VFs), as well as MGEs including plasmids, integrons and transposons.

## Results and Discussion

### Analysis of the plasmid metagenome of activated sludge using high-throughput sequencing

In order to deeply explore the plasmid metagenome, we employed a plasmid purification kit and a Plasmid Safe DNase to concentrate the plasmids in the DNA samples. Quantitative real time PCR (qPCR) indicated that the level of tetracycline resistance gene type *tetG* increased from 1.1×10^5^ copies/ng of total DNA to 4.1×10^6^ copies/ng of plasmid DNA after being purified by QIAGEN Plasmid Purification Kit and treated by Plasmid Safe DNase (Figure S1), indicating that the plasmids DNA were concentrated by about 37 fold after the treatments since the gene *tetG* is found to be only located on MGEs, such as plasmids (see Belgian Biodafty Server - Antibioresistance Archive, http://www.antibioresistance.be). However, PCRs demonstrated that 16S rRNA gene still occurred in the plasmid DNA samples, showing that chromosomal DNA contamination was not completely removed.

The plasmid metagenome of activated sludge was analyzed by Illumina Hiseq 2000 high-throughput sequencing, generating 11,550,210 clean reads comprising 1.2 Gb in total (Table S1). Annotation of all the reads showed that the majority was of bacterial origin while very small fractions came from fungi (548 reads, <0.005%) and protozoa (499 reads <0.005%). Nearly half (44.8%) of the Illumina reads was well assembled into a total of 4,641 contigs of over 500 bp, with N50 length of 3.0 kb and the total contig length of 7.1 Mb (Table S1). Qin et al. [20] established a human gut microbial gene catalogue by high-throughput sequencing and 42.7% of the Illumina reads was assembled into contigs of over 500 bp with an N50 length of only 2.2 kb. Comparatively, in this study more reads were well assembled and longer contigs were generated, indicating the validity of the high-throughput sequencing reads. In this study, MetaGene analyses revealed a total of 9,315 predicted open reading frames (ORFs) in the contigs longer than 100 bp (Table S2). They occupied 92.0% of the contigs, which is comparable to that in the human gut environments [20]. About 61.4% of the ORFs appeared incomplete, possibly due to the small size of the contigs (N50 of 3.0 kb). Among these ORFs, 25.7% and 67.7% (Table S3) could be annotated against the databases of KEGG (Kyoto Encyclopedia of Genes and Genomes) Pathway (Figure S2) and eggNOG (evolutionary genealogy of genes: Non-supervised Orthologous Groups) (Figure S3), respectively. Species annotation of ORFs showed that the bacterial community was dominated by *Actinobacteria*, *Chloroflexi*, *Proteobacteria*, *Bacteroidetes*, and *Firmicutes* (Figure S4), which was supported by our previous Roche 454-pyrosequencing results indicating that these five phyla dominated in activated sludge of Shatin STP [21]. Recently, a pyrosequencing study also indicated that the sediments of Indian and Sweden rivers contaminated by antibiotics dominantly contained *Proteobacteria*, *Bacteroidetes* and *Firmicutes* [17].

### Mapping of high-throughput sequencing reads to NCBI Plasmid Genome Database

**Figure 1:**
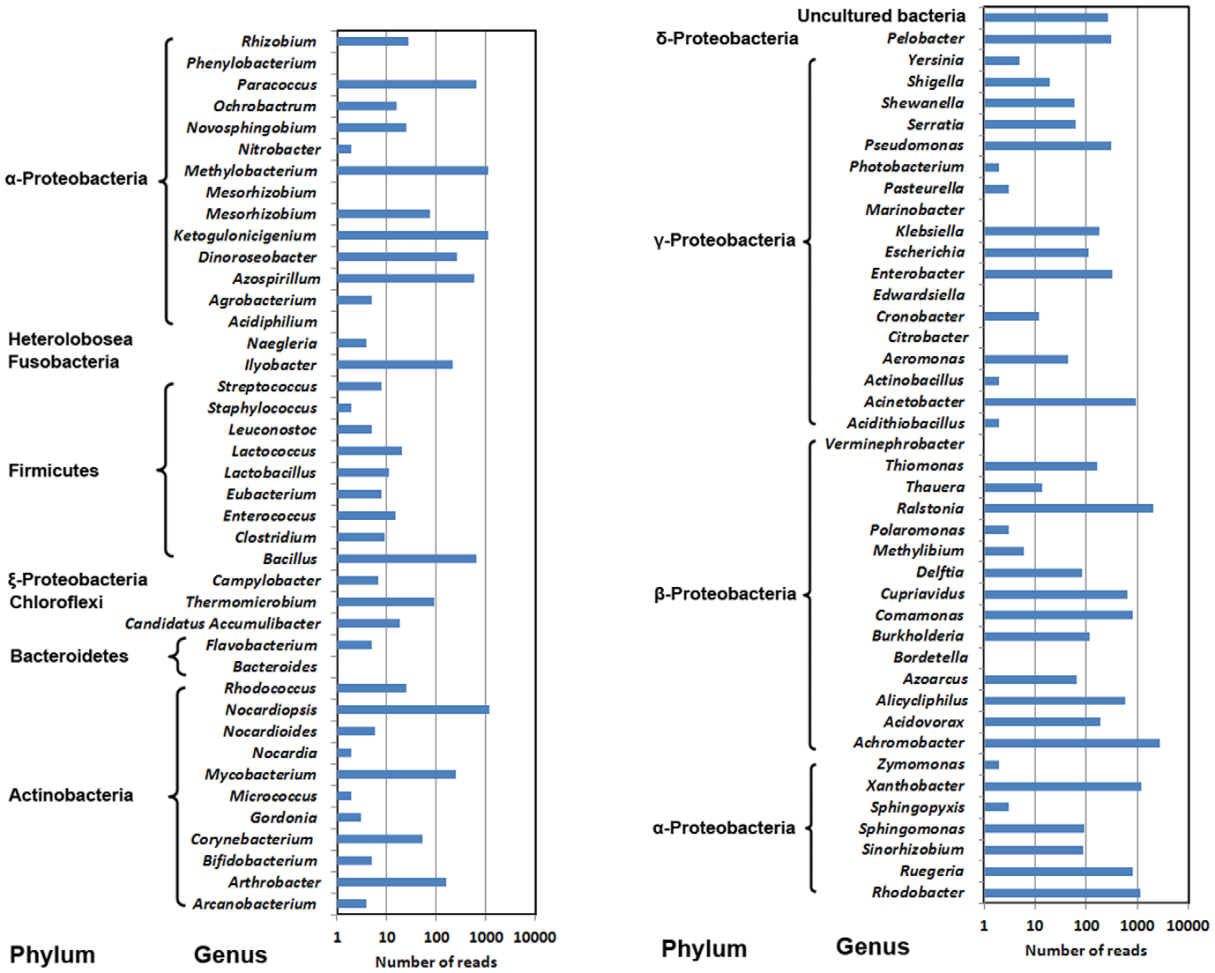
Abundances of bacterial hosts of plasmids in activated sludge of Shatin STP.

Plasmids are considered as the main vehicle for intercellular horizontal transfer of genetic materials and we therefore searched the metagenome for plasmid-associated DNA. Mapping all the reads to NCBI Plasmid Genome Database revealed that the number of matched reads ranged from 4,588 to 122,427 under different hit lengths (50–100 bp) and similarities (90%–100%) (Figure S5). In this study, we adopted strict cut-off criteria as sequence identity above 95% and hit length at least 90 bp [17]. As a result, a total of 20,363 reads (about 0.18%) and 130 contigs (2.80%) matched 307 different plasmids deposited in the database (Tables S4 and S5). These plasmids had various bacterial hosts affiliated to 83 genera, most of which belonged to *Proteobacteria* (Figure 1), which was confirmed by the species annotation against the NCBI non-redundant protein (NR) database (Figure S4). Among the identified plasmids, pA81 (2,513 reads) and pGMI1000MP (1,821 reads) had the highest abundances in the STP. Plasmid pA81 was isolated from the haloaromatic acid-degrading bacterium *Achromobacter xylosoxidans* and its complete 98,192-bp sequence contained 103 ORFs mostly encoding (halo)aromatic compound degradation or heavy metal resistance determinants [22]. pGMI1000MP is a megaplasmid (2,094,509 bp) often harbored in *Ralstonia solanacearum* [23], which accounts for its high abundance in the sludge metagenome. Several plasmids identified in this study, e.g. pB10 (67 reads), pTB11 (56 reads), pRSB101 (22 reads), pB4 (10 reads), pB3 (8 reads) and pGNB2 (1 read), were previously isolated from activated sludge of STPs and were proved to carry various ARGs, integrons or transposons [2], [3], [13], [14], [24], [25].

**Figure 2:**
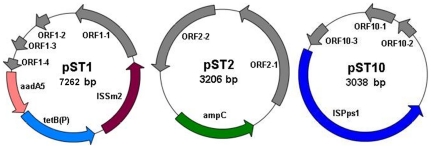
Open reading frames (ORFs) on the three plasmids detected in activated sludge of Shatin STP. pST1 structure was identified by assembling the sequencing reads using SOAPdenovo (BGI, Shenzhen, China). pST2 and pST10 were cloned with culture-independent transposon aided capture system and DNA sequencing were conducted applying the primer walking strategy.

The sequencing reads of one contig was well assembled to generate a previously undescribed plasmid (pST1), judging from a 26-bp overlap on both end of the contig sequence. BLAST searches demonstrate that the overlap fragment is completely identical to the gene encoding replication initiation protein on *Aeromonas hydrophila* plasmid pRA3 (Accession No. DQ401103.1) and *Aeromonas punctata* plasmid pFBAOT6 (Accession No. CR376602.1). Plasmid pST1 contained a tetracycline resistance gene (*tetB(P)*), an aminoglycoside resistance gene (*aadA5*) and a transposon (*ISSm2*) (Figure 2). The hypothesis of this plasmid gene map was supported by previous studies indicating that IS*Sm2* was similar to IS*Sm1* in terms of DNA sequence [26] and often located just upstream of the aminoglycoside resistance gene encoding acetyltransferase, such as *aadA5* [27].

### Isolation of novel plasmids from activated sludge microbiome using TRACA method

**Figure 3:**
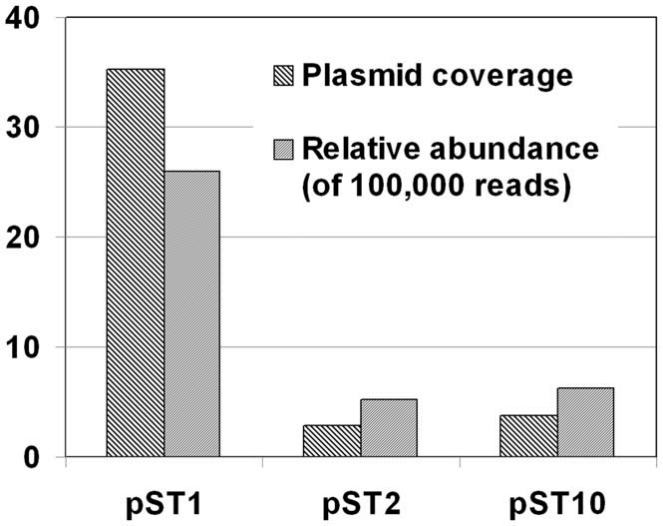
Relative abundances of the three plasmids in activated sludge of Shatin STP. The relative abundance is given in relation to the total number of sequencing reads and the plasmid coverage was estimated by counting the number of reads aligned at each base.

In order to acquire more novel plasmids, the TRACA system was used to isolate plasmids from extracted DNA of the activated sludge. Two plasmids (pST2 and pST10) were acquired and their sizes were 3,206 bp (pST2) and 3,038 bp (pST10), respectively (Figure 2). Genes assigned putative functions were identified on both plasmids along with ORFs of unknown function, many of which exhibited no significant homology to sequences available in the databases (Figure 2). This result indicates that some uncharacterized activities/functions are encoded by plasmids in the activated sludge community. Alignment of all the sequencing reads to the DNA sequences of pST1, pST2, and pST10 showed that the three plasmids were prevalent in the STP with high relative abundance and coverage (Figure 3).

For plasmid pST2, the only known putative function encoded by this plasmid was β-lactam resistance (Figure 2). The gene *amp*C encoding β-lactamase has been detected in the microbial isolates from wastewater, surface water, and even drinking water films [28]. This β-lactam resistance gene often coexists with other ARGs and can also be carried on MGEs, increasing the possibility of multidrug resistance and environmental dissemination [9], [12]. Plasmid pST10 was also predicted to harbor an ORF encoding a type of transposase (IS*Pps1*) (Figure 2) that is often present in *Pseudomonas pseudoalcaligenes* (accession number: AF028594) and *Acidovorax avenae* (accession number: AF086815).

### Presence of antibiotic resistance genes in the plasmid metagenome

**Figure 4:**
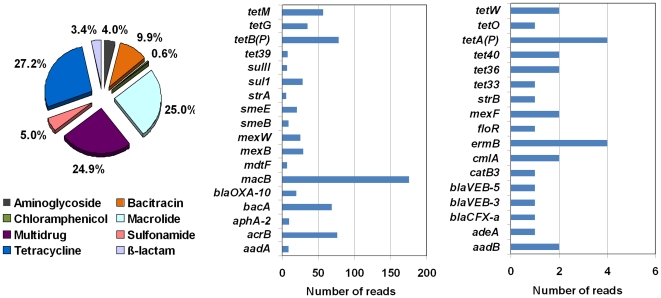
Relative abundances of various ARGs in activated sludge of Shatin STP. The relative abundance is given in relation to the total number of ARG-matched reads.

To characterize the activated sludge resistome in detail, we searched the plasmid metagenome for signatures of known ARGs in Antibiotic Resistance Database (ARDB) consisting of 23,137 sequences of 380 ARGs encoding resistance to 249 antibiotics [29]. A total of 699 reads (about 0.007%) and 3 contigs (0.06%) were assigned to the known antibiotic resistances (35 ARGs). The total abundance of ARGs in plasmids of activated sludge was relatively lower than those of antibiotic-contaminated sediments (0.02%–1.71%) [17]. The types of ARGs identified in this study were relatively less than the plasmid metagenome data that were previously obtained by 454-pyrosequencing for the sludge bacteria cultured with 12 types of different antibiotics individually [15], [16]. The reason that more ARGs were identified in the previous studies may be the magnification of antibiotic resistances in the sludge cultured with different types of antibiotics [15], [16], thus this culture-dependent method cannot reflect the actual abundances of different ARGs since many trace-level genes have been amplified to reach detectable levels. Additionally, to ensure the accuracy of the sequence annotation using BLAST, we adopted a comparatively more stringent cut-off (sequence identity >90%; hit length >25 AA), which may also lower down the abundances of ARGs detected in this study. Our results demonstrated that the ARGs coding for tetracycline (27.2%), macrolide (25.0%) and multidrug (24.9%) resistances were highly rich in the activated sludge (Figure 4). Recent studies have indicated that various tetracycline resistance genes (*tet*) often occur in STPs with high abundances [11], [30], [31]. Among the *tet* genes, *tetB(P)*, *tetM* and *tetG* had the highest abundance (Table S6). However, our previous study using qPCR demonstrated that *tetA*, *tetC*, *tetG*, *tetM* and *tetA(P)* were relatively more abundant in this STP [11]. This difference might be due to the PCR bias limited by the specificity of primer sets and the temporal variation of ARGs in STPs [31]. The gene *macB* was the most abundant ARG (175 reads) (Figure 4), which encodes macrolide-specific efflux system (Table S6). The *mac* genes can be easily transferred from one host to another [32], since they are usually acquired by mobile elements, such as plasmids [33] and transposons [34].

### Presence of integrons, transposons and virulence factors in the plasmid metagenome

The mobility of ARGs depends on the MGEs, such as plasmids, integrons and transposons [8], [35]. Totally, 338 reads (0.003%) and 4 contigs (0.09%) could be assigned to known integronase genes and gene cassettes (Table S7) deposited in INTEGRALL database [36]. Class 1, 2 and 3 integrons occupied 92.6% (313 reads), 5.0% (17 reads) and 0.6% (2 reads) of the alignment hits, respectively. This result was supported by previous studies indicating that class 1 was the most abundant among the several identified integrons in STPs [10], [37]. Gene cassettes identified in this study contained various ARGs encoding resistances to β-lactam (*ampC*, *bla_VEB-3_*, *bla_VIM-2_* and *bla_IMP-1_*), aminoglycoside (*aacA4*, *aadA1*, *aadA2*, *aadA2b* and *aadA24*), sulphonamides (*sulI*), trimethoprim (*dfrA1*) and quaternary ammonium compounds (*qacEΔ1*). Alignment against the transposon and insertion sequence database ISfinder [38] demonstrated that a total of 4,013 reads (0.035%) and 37 contigs (0.80%) could match known transposons or insertion sequences (Table S8). Among the identified insertion sequences present in the activated sludge, IS*Pps1* (1,048 reads) had the highest abundance, followed by IS*Sm2* (679 reads) and IS*Aba3* (306 reads). This was further confirmed by the presence of IS*Pps1* on plasmid pST10 and IS*Sm2* on plasmid pST1 since the two high-copy plasmids were isolated from the activated sludge.

Pathogenic bacteria are able to induce different diseases by expression of different combinations of VFs, which is often mediated by MGEs [39], [40]. We therefore further compared the plasmid metagenome against the VFs database [41] in order to uncover the pathogenic information in activated sludge. As a result, 1,237 reads (0.011%) and 10 contigs (0.22%) were assigned to known virulence factors. Among the identified VFs, VFG1405 (233 reads) and VFG1031 (164 reads) had relatively higher abundance (Table S9). VFG1031 encoding transposase Tn*pA* is often carried by pathogen *Shigella flexneri* associated with multiple antibiotic resistances [42]. Virulence factor VFG1028 (class 1 integron carried by *S*. *flexneri*) also had high abundance, which usually includes β-lactamase (*bla_OXA-30_*) and streptomycin adenyltransferase (*aadA1*) genes in their gene cassettes [42]. This study demonstrated that the TRACA system in combination with high-throughput sequencing and metagenomics formed a reliable way to reveal real situation of ARGs, plasmids, integrons, transposons and VFs in activated sludge of STPs. However, the plasmid metagenome obtained in this study was contaminated by chromosomal DNA although the plasmids portion was significantly increased in the extracted DNA after being purified by QIAGEN Plasmid Purification Kit and subsequently treated by the Plasmid Safe DNase to remove the remaining linear DNA. Furthermore, due to the high biodiversity in activated sludge (more than 2,000 OTUs at 3% cut-off level) [21], the 1.2 billion DNA bases generated within this study maybe only cover a fraction of the whole metagenome [43], [44], which thus affects the statistical power of detecting ARGs and MGEs with lower abundance and complicates the assembly of larger plasmids which are generally present in lower copy numbers [17]. In order to comprehensively and deeply characterize the metagenome and the associated resistome, it is necessary to increase the sequencing depth by applying high-throughput DNA sequencing techniques and update *in silico* analysis methods of metagenomics data.

Our results show that various MGEs are present in STPs and these elements may play important roles in horizontal transfer of various ARGs among the bacterial species. This highlights the fact that STPs serve as potential recruitment pools for antibiotic resistant bacteria. ARGs may enter into water or soil environments along with STP effluent and the agricultural application of sludge [1]. The appearance of ARGs in STPs increases surveillance for risk assessment and prevention strategies to protect public health, since the sludge with high microbial diversity and biomass facilitates the horizontal transfer of antibiotic resistances [10], [12]. Furthermore, long-term exposure to antibiotics in wastewater at subinhibitory or even lower levels may favor horizontal transfer of ARGs in activated sludge [15]. Some efforts therefore should be motivated to reduce the possibility of ARGs entering into and spread in the environments. The most effective and direct approach to prevent the environment from antibiotic resistant bacteria is reasonable use of antibiotics in human health protection and veterinary application.

## Materials and Methods

### Sampling and DNA extraction

Activated sludge samples were collected from Shatin Sewage Treatment Plant (Hong Kong, China) at three times, separately on August 17, October 12 and November 18 of 2010. Details about the STP were previously described [10], [11]. The sludge was centrifuged to collect approximately 200 mg of the pellet for total genomic DNA extraction using the FastDNA Soil Kit (MP Biomedicals, CA, USA). After DNA concentration was determined by microspectrophotometry (NanoDrop® ND-1000, NanoDrop Technologies, Willmington, DE), equal mass of the total DNA extracted from the above three sludge samples collected at each time point was mixed to minimize the temporal variation. About 100 µg of the mixed DNA was further purified by using QIAGEN Plasmid Mini Kit (QIAGEN SCIENCES INC, Germantown, MD, USA) to concentrate plasmids. In order to remove remaining sheared genomic DNA, approximately 1 µg of plasmid DNA was treated with ATP-dependant Plasmid Safe DNase (Epicentre, USA) according to manufacturer's instructions. Plasmid Safe digests were incubated overnight at 37°C and subsequently inactivated by heating at 70°C for 30 min followed by chilling on ice. The obtained DNA samples were then used for Illumina sequencing and plasmid capture reactions. In order to evaluate the plasmid enrichment assay, qPCR was used to determine relative abundance of the well-known plasmid borne *tetG* (tetracycline resistance gene) in the total DNA directly isolated from sludge and the concentrated plasmid metagenome DNA. The copy number was normalized to DNA mass and the qPCR procedures were previously described in detail [11]. PCRs of 16S rRNA gene were conducted to check the contamination of chromosomal DNA in the plasmid metagenome by using the primer set of 341F and 518R according to a previous study [45].

### Isolation of novel plasmids

Plasmids were isolated from Plasmid Safe treated metagenomic preparations using the TRACA system according to Jones et al. [46]. TRACA reactions were set up using the EZ-*Tn5* ^TM^ <*ori*V/KAN-2> Insertion Kit (Epicentre, USA). Each reaction was composed of Plasmid Safe digested metagenomic DNA (0.5 µg), transposase (1 U), 10× EZ-*Tn5* reaction buffer (2 µl) and brought to a final volume of 20 µl by adding sterile pure water. Reactions were conducted for 2 h at 37°C and stopped by adding EZ-*Tn5* stop buffer (2 µl) and heating at 70°C for 10 min. Then, reaction mixtures were applied to transform TransforMax™ EPI300™ Chemically Competent *E. coli* (Epicentre, USA) according to the manufacturer's introduction. Transformants were selected on LB agar supplemented with 50 mg/l kanamycin and cultured in Growth Media (Epicentre, USA) to amplify low-copy plasmid clones. Plasmids with transposon insert were extracted and purified using MiniBEST Plasmid Purification Kit (TaKaRa, Japan). The plasmids acquired were sequenced by BGI (Shenzhen, China) using the primer walking strategy.

### High-throughput sequencing of activated sludge metagenome

High-throughput sequencing was performed by BGI (Shenzhen, China) using Illumina Hiseq 2000. The sequencing strategy was index PE101+8+101 cycle (Paired End sequencing, 101-bp reads and 8-bp index sequence). More than 1 Gb of sequences was generated for the sludge DNA sample. The raw reads containing three or more “N” or contaminated with adaptors were removed and the remaining clean reads (98.9%) were used for further analysis. The metagenomic data have been deposited at NCBI Sequence Read Archive under accession number SRP007256.1. Nucleotide sequences of the plasmids were deposited at GenBank under accession numbers JN098513 - JN098515.

### Computational analysis and bioinformatics

All sequencing reads were assembled by using SOAPdenovo (BGI, Shenzhen, China) with Kmer at 55. The contigs were compared against the non-redundant protein database at NCBI GenBank using BLAST (E-value cut-off of 10^−10^). MetaGene program [47] was used to predict ORFs in the contigs. Functional annotation was conducted by comparing the contigs against KEGG [48] and eggNOG [49] databases (E-value cut-off of 10^−10^).

In order to characterize the plasmid metagenome, all reads were aligned to plasmid genome sequences available in the NCBI RefSeq database (2,408 sequences). The alignment was performed using BLAST against the known plasmids. BLAST hits (blastn) were determined for the alignments with a nucleotide sequence identity above 95% over a length of at least 90 bp. Plasmid coverage of pST1, pST2 and pST10 was calculated by counting the number of reads aligned at each base according to Kristiansson et al. [17]. The obtained contigs were subject to assembly of novel plasmids by concatenating overlapping in a step-wise fashion (minimum 95% sequence identity) until the fragment was found to be circular. After assembly, the plasmids were manually edited for quality control and ORFs were predicted using NCBI ORF Finder. Genetic maps of the plasmids were constructed using Plasm software (ver 2.0.4.29) (http://biofreesoftware.com).

A database of resistances genes was created from all sequences in ARDB [29]. A read was annotated as a resistance gene according to its best BLAST hit (blastx) with a given threshold of amino acid sequence identity above 90% and the alignment at least 25 amino acids [17]. To characterize the mobile elements in the activated sludge in detail, we searched the metagenomes for signatures of known integrons and transposons in reference databases. A database of integrons was created from the nucleotide sequences for all integrases available in the database INTEGRALL (1,447 integrase genes and 8,053 gene cassettes) [36]. All sequences of transposons from the ISfinder [38] were downloaded (2,578 sequences, 22 families of insertion sequences) to develop a transposon database. A read was annotated as an integron or insertion sequence if the BLAST hit (blastn) had a sequence identity of more than 90% over an alignment of at least 50 bases [17].

DNA sequences of virulence factors (VFs) (2,312 sequences) were downloaded from VFDB [41] for the alignment of all reads against the VFs database. A read was assigned to a VF if the BLAST hit (blastn) had an identity of over 90% and an alignment of at least 50 bases [50].

## Supporting Information

Table S1 **Assembling analysis of high-throughput sequencing reads in plasmid metagenome of activated sludge with the optimal Kmer at 55.**
(DOC)

Table S2 **Prediction of open reading frames (ORFs) using Metagene Annotator Software.**
(DOC)

Table S3 **Statistical analysis on functional classification and species annotation of the available open reading frames according to different databases.**
(DOC)

Table S4 **Matched assembled contigs of plasmids in the activated sludge of Shatin STP.**
(DOC)

Table S5 **Matched high-throughput sequencing reads of plasmids in the activated sludge of Shatin STP.**
(DOC)

Table S6 **Matched high-throughput sequencing reads of antibiotic resistance genes (ARGs) in the activated sludge against Antibiotic Resistance Database (ARDB).**
(DOC)

Table S7 **Matched high-throughput sequencing reads of integrons and gene cassettes in the activated sludge of Shatin STP.**
(DOC)

Table S8 **Matched high-throughput sequencing reads of insertion sequence common region transposases in plasmid metagenome of activated sludge of Shatin STP.**
(DOC)

Table S9 **Matched high-throughput sequencing reads of virulence factors in plasmid metagenome of activated sludge of Shatin STP.**
(DOC)

Figure S1 **Calibration curves of quantitative real-time PCR for tetracycline resistance gene** ***tetG*** **generated with serial dilutions of plasmid vector carrying the target gene** ***tetG*** **(A) and comparison of** ***tetG*** **abundance in the environmentally total DNA and the concentrated plasmid metagenome isolated from activated sludge (B).**
(TIF)

Figure S2 **KEGG (Kyoto Encyclopedia of Genes and Genomes) pathway classification of plasmid metagenome of the activated sludge in Shatin STP (E-value cut off of 10^−10^).**
(TIF)

Figure S3 **Functional classification of plasmid metagenome in the activated sludge of Shatin STP against evolutionary genealogy of genes:** Non-supervised Orthologous Groups (eggNOG) databases (E-value cut-off of 10^−10^).
(TIF)

Figure S4 **Species annotation of ORFs of plasmid metagenome in the activated sludge of Shatin STP against the non-redundant protein database at NCBI GenBank (E-value cut-off of 10^−10^).**
(TIF)

Figure S5 **Number of matched high-throughput sequencing reads against NCBI plasmid genome database by using different cut-offs (hit length and sequence identity).** The number of hits has significant negative correlation with both hit length and sequence identity.
(TIF)
