# Supplementary material for: Plasmid Metagenome Reveals High Levels of Antibiotic Resistance Genes and Mobile Genetic Elements in Activated Sludge
Source: PLoS One. 2011 Oct 10;6(10):e26041. doi: 10.1371/journal.pone.0026041 (PMC3189950; doi:10.1371/journal.pone.0026041)
Supplement: Table S1 — Assembling analysis of high-throughput sequencing reads in plasmid metagenome of activated sludge with the optimal Kmer at 55. (DOC) [file pone.0026041.s001.doc]

| Kmer | ContigNum | ContigLen (bp) | N50 (bp) | N90 (bp) | MaxLen (bp) | MinLen (bp) |
| --- | --- | --- | --- | --- | --- | --- |
| K53 | 5,247 | 7,675,472 | 2,639 | 582 | 37,294 | 500 |
| **K55** | **4,641** | **7,051,537** | **2,998** | **587** | **37,480** | **500** |
| K57 | 4,328 | 6,360,389 | 2,502 | 587 | 29,708 | 500 |
