# Supplementary material for: Plasmid Metagenome Reveals High Levels of Antibiotic Resistance Genes and Mobile Genetic Elements in Activated Sludge
Source: PLoS One. 2011 Oct 10;6(10):e26041. doi: 10.1371/journal.pone.0026041 (PMC3189950; doi:10.1371/journal.pone.0026041)
Supplement: Table S2 — Prediction of open reading frames (ORFs) using Metagene Annotator Software. (DOC) [file pone.0026041.s002.doc]

| Number of raw reads | 11,683,136 | Number of clean reads | 11,550,210 |
| --- | --- | --- | --- |
| Number of ORFs | 9,351 | Complete ORFs (%) | 38.65 |
| Total length of ORFs (bp) | 6,302,544 | Fragmental ORFs (%) | 61.35 |
| Average length of ORFs (bp) | 674 | Contig coverage (%) | 92 |
