# Supplementary material for: Plasmid Metagenome Reveals High Levels of Antibiotic Resistance Genes and Mobile Genetic Elements in Activated Sludge
Source: PLoS One. 2011 Oct 10;6(10):e26041. doi: 10.1371/journal.pone.0026041 (PMC3189950; doi:10.1371/journal.pone.0026041)
Supplement: Table S3 — Statistical analysis on functional classification and species annotation of the available open reading frames according to different databases. (DOC) [file pone.0026041.s003.doc]

|  | Sample | ST plasmid |
| --- | --- | --- |
|  | Total ORF number | 9,351 |
| Non-redundant protein database at NCBI GenBank | Unknown | 12.37% |
|  | Unclassified | 11.83% |
|  | Classified | 65.03% |
| eggNOG | Unannotated | 32.26% |
|  | Annotated | 67.74% |
| KEGG Orthology | Unannotated | 41.73% |
|  | Annotated | 58.27% |
| KEGG Pathway | Unannotated | 74.30% |
|  | Annotated | 25.70% |
