# Supplementary material for: Plasmid Metagenome Reveals High Levels of Antibiotic Resistance Genes and Mobile Genetic Elements in Activated Sludge
Source: PLoS One. 2011 Oct 10;6(10):e26041. doi: 10.1371/journal.pone.0026041 (PMC3189950; doi:10.1371/journal.pone.0026041)
Supplement: Table S4 — Matched assembled contigs of plasmids in the activated sludge of Shatin STP. (DOC) [file pone.0026041.s004.doc]

| Accession number | Bacterial hosts | Identity (%) ≥ | Hit length (bp) ≥ | E value ≤ | Number of contigs |
| --- | --- | --- | --- | --- | --- |
| NC_003296.1 | *Ralstonia solanacearum* | 95 | 107 | 1.00E-42 | 10 |
| NC_003350.1 | *Pseudomonas putida* | 96 | 165 | 3.00E-72 | 3 |
| NC_004604.2 | *Bacillus megaterium* | 96 | 111 | 4.00E-46 | 4 |
| NC_004956.1 | *Pseudomonas* sp. | 100 | 219 | < 1.0E-100 | 1 |
| NC_005241.1 | *Cupriavidus necator* | 97 | 156 | 1.00E-73 | 1 |
| NC_006352.1 | *Uncultured bacterium in activated sludge* | 100 | 182 | 5.00E-99 | 1 |
| NC_006362.1 | *Nocardia farcinica* | 95 | 101 | 2.00E-38 | 1 |
| NC_006823.1 | *Azoarcus* sp. | 100 | 470 | < 1.0E-100 | 1 |
| NC_007974.2 | *Cupriavidus metallidurans* | 95 | 190 | 3.00E-82 | 2 |
| NC_008043.1 | *Escherichia coli* | 95 | 162 | 5.00E-68 | 12 |
| NC_008055.1 | Uncultured bacterium in freshwater | 99 | 454 | < 1.0E-100 | 1 |
| NC_008573.1 | *Shewanella* sp. | 100 | 167 | 4.00E-90 | 1 |
| NC_008608.1 | *Pelobacter propionicus* | 98 | 343 | < 1.0E-100 | 9 |
| NC_008712.1 | *Arthrobacter aurescens* | 100 | 671 | < 1.0E-100 | 2 |
| NC_009429.1 | *Rhodobacter sphaeroides* | 95 | 162 | 5.00E-68 | 12 |
| NC_009651.1 | *Klebsiella pneumoniae* | 100 | 134 | 2.00E-70 | 1 |
| NC_009717.1 | *Xanthobacter autotrophicus* | 99 | 551 | < 1.0E-100 | 1 |
| NC_009753.1 | *Paracoccus methylutens* | 100 | 1425 | < 1.0E-100 | 2 |
| NC_010404.1 | *Acinetobacter baumannii* | 95 | 310 | < 1.0E-100 | 7 |
| NC_010488.1 | *Escherichia coli* | 98 | 202 | 6.00E-99 | 1 |
| NC_010510.1 | *Methylobacterium radiotolerans* | 95 | 232 | < 1.0E-100 | 10 |
| NC_010605.1 | *Acinetobacter baumannii* | 96 | 424 | < 1.0E-100 | 2 |
| NC_010606.1 | *Acinetobacter baumannii* | 99 | 592 | < 1.0E-100 | 2 |
| NC_010935.1 | *Comamonas testosteroni* | 95 | 164 | 3.00E-69 | 12 |
| NC_011961.1 | *Thermomicrobium roseum* | 96 | 94 | 6.00E-37 | 1 |
| NC_012556.1 | *Enterobacter cloacae* | 100 | 1095 | < 1.0E-100 | 1 |
| NC_012849.1 | *Ralstonia pickettii* | 100 | 103 | 1.00E-51 | 1 |
| NC_013859.1 | *Azospirillum* sp. | 95 | 106 | 6.00E-42 | 3 |
| NC_014005.1 | *Sphingobium japonicum* | 100 | 743 | < 1.0E-100 | 1 |
| NC_014155.1 | *Thiomonas intermedia* | 97 | 321 | < 1.0E-100 | 4 |
| NC_014167.1 | *Corynebacterium resistens* | 100 | 182 | 5.00E-99 | 1 |
| NC_014211.1 | *Nocardiopsis dassonvillei* | 95 | 125 | 7.00E-51 | 6 |
| NC_014309.1 | *Ralstonia solanacearum* | 96 | 116 | 1.00E-47 | 1 |
| NC_014621.1 | *Ketogulonicigenium vulgare* | 95 | 173 | 1.00E-74 | 9 |
| NC_014633.1 | *Ilyobacter polytropus* | 96 | 112 | 2.00E-45 | 2 |
| NC_014911.1 | *Alicycliphilus denitrificans* | 96 | 290 | < 1.0E-100 | 1 |
