# Supplementary material for: Plasmid Metagenome Reveals High Levels of Antibiotic Resistance Genes and Mobile Genetic Elements in Activated Sludge
Source: PLoS One. 2011 Oct 10;6(10):e26041. doi: 10.1371/journal.pone.0026041 (PMC3189950; doi:10.1371/journal.pone.0026041)
Supplement: Table S5 — Matched high-throughput sequencing reads of plasmids in the activated sludge of Shatin STP. (DOC) [file pone.0026041.s005.doc]

| Accession number | Bacterial host | Plasmid name | Identity (%) ≥ | Hit length (bp) ≥ | E value ≤ | Number of reads |
| --- | --- | --- | --- | --- | --- | --- |
| NC_008055.1 | Uncultured bacterium in freshwater | QKH54 | 95 | 100 | 2.0E-26 | 65 |
| NC_008147.1 | *Mycobacterium* sp. | plasmid1 | 97 | 92 | 2.0E-38 | 1 |
| NC_002143.1 | *Comamonas testosteroni* | pPT1 | 99 | 100 | 5.0E-48 | 2 |
| NC_002377.1 | *Agrobacterium tumefaciens* | Ti | 97 | 99 | 1.0E-42 | 2 |
| NC_002525.1 | *Escherichia coli* | R721 | 99 | 100 | 5.0E-48 | 3 |
| NC_000923.1 | *Acinetobacter* sp. | pRAY | 96 | 100 | 8.0E-41 | 3 |
| NC_008242.1 | *Mesorhizobium* sp. | plasmid 1 | 95 | 100 | 2.0E-38 | 59 |
| NC_008243.1 | *Mesorhizobium* sp. | plasmid 2 | 99 | 92 | 3.0E-43 | 1 |
| NC_008244.1 | *Mesorhizobium* sp. | plasmid 3 | 99 | 94 | 2.0E-44 | 1 |
| NC_008246.1 | *Sphingobium yanoikuyae* | pYAN-1 | 100 | 95 | 2.0E-47 | 1 |
| NC_008269.1 | *Rhodococcus jostii* | pRHL1 | 95 | 100 | 2.0E-38 | 8 |
| NC_008272.1 | *Escherichia coli* | pKJK5 | 96 | 93 | 1.0E-36 | 6 |
| NC_008308.1 | *Sphingomonas* sp. | pCAR3 | 95 | 100 | 2.0E-38 | 11 |
| NC_006827.2 | *Enterococcus faecalis* | pCF10 | 100 | 100 | 2.0E-50 | 4 |
| NC_008010.2 | *Deinococcus geothermalis* | pDGEO01 | 95 | 100 | 2.0E-38 | 59 |
| NC_001735.4 | *Enterobacter aerogenes* | R751 | 100 | 100 | 2.0E-50 | 1 |
| NC_008357.1 | *Pseudomonas aeruginosa* | pBS228 | 98 | 92 | 8.0E-41 | 2 |
| NC_008330.1 | Uncultured bacterium in soil | pLB1 | 95 | 100 | 2.0E-38 | 13 |
| NC_008385.1 | *Burkholderia cepacia* | plasmid 1 | 95 | 100 | 2.0E-26 | 33 |
| NC_008445.1 | *Enterococcus faecalis* | pRE25 | 99 | 101 | 3.0E-46 | 1 |
| NC_008459.1 | *Bordetella pertussis* | pBP136 | 99 | 100 | 5.0E-48 | 1 |
| NC_008379.1 | *Rhizobium leguminosarum* | pRL9 | 96 | 93 | 1.0E-36 | 2 |
| NC_008382.1 | *Rhizobium leguminosarum* | pRL7 | 95 | 100 | 2.0E-38 | 2 |
| NC_008384.1 | *Rhizobium leguminosarum* | pRL11 | 96 | 92 | 5.0E-36 | 1 |
| NC_008505.1 | *Lactococcus lactis* | plasmid 3 | 96 | 94 | 3.0E-37 | 7 |
| NC_008506.1 | *Lactococcus lactis* | plasmid 4 | 99 | 100 | 5.0E-48 | 6 |
| NC_007682.3 | *Escherichia coli* | pMUR050 | 100 | 96 | 5.0E-48 | 1 |
| NC_008573.1 | *Shewanella* sp. | plasmid 1 | 95 | 100 | 2.0E-38 | 59 |
| NC_008608.1 | *Pelobacter propionicus* | pPRO2 | 95 | 100 | 2.0E-38 | 306 |
| NC_008613.1 | *Photobacterium damselae* | pP91278 | 100 | 100 | 2.0E-50 | 1 |
| NC_008612.1 | *Photobacterium damselae* | pP99-018 | 100 | 100 | 2.0E-50 | 1 |
| NC_008688.1 | *Paracoccus denitrificans* | plasmid 1 | 95 | 100 | 2.0E-38 | 15 |
| NC_008697.1 | *Nocardioides* sp. | pNOCA01 | 95 | 100 | 2.0E-38 | 6 |
| NC_008703.1 | *Mycobacterium* sp. | pMKMS01 | 95 | 100 | 2.0E-38 | 152 |
| NC_008704.1 | *Mycobacterium* sp. | pMKMS02 | 95 | 100 | 2.0E-38 | 6 |
| NC_008712.1 | *Arthrobacter aurescens* | TC1 | 95 | 100 | 2.0E-38 | 161 |
| NC_008739.1 | *Marinobacter aquaeolei* | pMAQU02 | 96 | 96 | 2.0E-38 | 1 |
| NC_008765.1 | *Acidovorax* sp. | pAOVO01 | 95 | 100 | 2.0E-26 | 89 |
| NC_008766.1 | *Acidovorax* sp. | pAOVO02 | 95 | 100 | 2.0E-26 | 82 |
| NC_008757.1 | *Polaromonas naphthalenivorans* | pPNAP01 | 95 | 100 | 2.0E-38 | 1 |
| NC_008760.1 | *Polaromonas naphthalenivorans* | pPNAP04 | 98 | 96 | 3.0E-43 | 1 |
| NC_008771.1 | *Verminephrobacter eiseniae* | pVEIS01 | 96 | 100 | 8.0E-41 | 1 |
| NC_008790.1 | *Campylobacter jejuni* | pTet | 97 | 95 | 3.0E-40 | 3 |
| NC_008821.1 | *Enterococcus faecium* | pVEF2 | 97 | 100 | 3.0E-43 | 2 |
| NC_008826.1 | *Methylibium petroleiphilum* | RPME01 | 95 | 100 | 2.0E-38 | 6 |
| NC_009083.1 | *Acinetobacter baumannii* | pAB1 | 95 | 100 | 2.0E-38 | 1 |
| NC_009084.1 | *Acinetobacter baumannii* | pAB2 | 99 | 95 | 5.0E-45 | 2 |
| NC_002682.1 | *Mesorhizobium loti* | pMLb | 96 | 100 | 8.0E-41 | 3 |
| NC_009131.1 | *Escherichia coli* | pLEW517 | 96 | 100 | 8.0E-41 | 5 |
| NC_009132.1 | *Escherichia coli* | pLEW517 | 96 | 100 | 8.0E-41 | 5 |
| NC_009133.1 | *Escherichia coli* | NR1 | 100 | 94 | 8.0E-47 | 2 |
| NC_009128.1 | *Corynebacterium* sp. | pLEW279a | 96 | 100 | 8.0E-41 | 6 |
| NC_009139.1 | *Yersinia ruckeri* | pYR1 | 98 | 97 | 8.0E-44 | 4 |
| NC_009141.1 | *Yersinia pestis* | pIP1202 | 100 | 96 | 5.0E-48 | 1 |
| NC_009140.1 | *Salmonella enterica* | pSN254 | 95 | 100 | 2.0E-38 | 15 |
| NC_009227.1 | *Burkholderia vietnamiensis* | pBVIE02 | 95 | 100 | 2.0E-38 | 9 |
| NC_002698.1 | *Shigella flexneri* | pWR501 | 97 | 100 | 3.0E-43 | 19 |
| NC_002679.1 | *Mesorhizobium loti* | pMLa | 95 | 100 | 2.0E-38 | 13 |
| NC_009339.1 | *Mycobacterium gilvum* | pMFLV01 | 95 | 100 | 2.0E-38 | 7 |
| NC_009341.1 | *Mycobacterium gilvum* | pMFLV03 | 97 | 100 | 5.0E-42 | 2 |
| NC_009349.1 | *Aeromonas salmonicida* | plasmid 4 | 96 | 92 | 5.0E-36 | 6 |
| NC_009350.1 | *Aeromonas salmonicida* | plasmid 5 | 96 | 92 | 5.0E-36 | 1 |
| NC_009426.1 | *Novosphingobium aromaticivorans* | pNL1 | 95 | 100 | 2.0E-38 | 25 |
| NC_009429.1 | *Rhodobacter sphaeroides* | pRSPA01 | 95 | 100 | 2.0E-38 | 1038 |
| NC_009430.1 | *Rhodobacter sphaeroides* | pRSPA02 | 95 | 100 | 2.0E-38 | 72 |
| NC_009435.1 | *Lactococcus lactis* | pGdh442 | 96 | 100 | 8.0E-41 | 3 |
| NC_009471.1 | *Acidiphilium cryptum* | pACRY05 | 98 | 97 | 8.0E-44 | 1 |
| NC_009507.1 | *Sphingomonas wittichii* | pSWIT01 | 95 | 100 | 2.0E-38 | 8 |
| NC_009508.1 | *Sphingomonas wittichii* | pSWIT02 | 96 | 90 | 7.0E-35 | 55 |
| NC_009620.1 | *Sinorhizobium medicae* | pSMED01 | 96 | 98 | 1.0E-39 | 2 |
| NC_009621.1 | *Sinorhizobium medicae* | pSMED02 | 95 | 100 | 2.0E-38 | 4 |
| NC_009622.1 | *Sinorhizobium medicae* | pSMED03 | 95 | 100 | 2.0E-38 | 1 |
| NC_003042.1 | *Clostridium perfringens* | pCP13 | 99 | 93 | 8.0E-44 | 2 |
| NC_009649.1 | *Klebsiella pneumoniae* | pKPN3 | 96 | 100 | 8.0E-41 | 2 |
| NC_009650.1 | *Klebsiella pneumoniae* | pKPN4 | 100 | 91 | 5.0E-45 | 2 |
| NC_009651.1 | *Klebsiella pneumoniae* | pKPN5 | 96 | 100 | 2.0E-38 | 4 |
| NC_009653.1 | *Klebsiella pneumoniae* | pKPN7 | 96 | 90 | 7.0E-35 | 1 |
| NC_009669.1 | *Ochrobactrum anthropi* | pOANT01 | 95 | 100 | 2.0E-38 | 13 |
| NC_009670.1 | *Ochrobactrum anthropi* | pOANT02 | 100 | 93 | 3.0E-46 | 1 |
| NC_009671.1 | *Ochrobactrum anthropi* | pOANT03 | 96 | 90 | 7.0E-35 | 2 |
| NC_009717.1 | *Xanthobacter autotrophicus* | pXAUT01 | 95 | 100 | 2.0E-38 | 1177 |
| NC_009739.1 | *Pseudomonas aeruginosa* | pMATVIM-7 | 96 | 100 | 8.0E-41 | 5 |
| NC_009751.1 | *Lactococcus lactis* | pK214 | 99 | 100 | 5.0E-48 | 2 |
| NC_009753.1 | *Paracoccus methylutens* | pMTH1 | 95 | 100 | 2.0E-38 | 634 |
| NC_009779.1 | *Cronobacter sakazakii* | pESA2 | 96 | 97 | 5.0E-39 | 12 |
| NC_009838.1 | *Escherichia coli* | pAPEC-O1-R | 96 | 100 | 8.0E-41 | 11 |
| NC_009955.1 | *Dinoroseobacter shibae* | pDSHI01 | 95 | 100 | 2.0E-38 | 63 |
| NC_009956.1 | *Dinoroseobacter shibae* | pDSHI02 | 96 | 91 | 2.0E-35 | 3 |
| NC_009957.1 | *Dinoroseobacter shibae* | pDSHI03 | 95 | 100 | 2.0E-38 | 103 |
| NC_009958.1 | *Dinoroseobacter shibae* | pDSHI04 | 95 | 100 | 2.0E-38 | 88 |
| NC_003122.1 | *Uncultured bacterium in soil* | pSB102 | 97 | 96 | 8.0E-41 | 2 |
| NC_009980.1 | *Salmonella enterica* | pMAK2 | 97 | 99 | 1.0E-42 | 1 |
| NC_009982.1 | *Salmonella enterica* | pMAK3 | 100 | 96 | 5.0E-48 | 1 |
| NC_009981.1 | *Salmonella enterica* | pMAK1 | 96 | 100 | 8.0E-41 | 5 |
| NC_010076.1 | *Bacillus thuringiensis* | pBtoxis | 98 | 97 | 8.0E-44 | 1 |
| NC_010119.1 | *Salmonella enterica* | pOU7519 | 100 | 94 | 8.0E-47 | 1 |
| NC_003037.1 | *Sinorhizobium meliloti* | pSymA | 95 | 100 | 2.0E-38 | 6 |
| NC_003078.1 | *Sinorhizobium meliloti* | pSymB | 95 | 100 | 2.0E-38 | 10 |
| NC_010189.1 | *Naegleria gruberi* | unnamed | 96 | 100 | 8.0E-41 | 2 |
| NC_010309.1 | *Acinetobacter venetianus* | pAV1 | 95 | 100 | 2.0E-38 | 18 |
| NC_010310.1 | *Acinetobacter venetianus* | pAV2 | 96 | 92 | 5.0E-36 | 27 |
| NC_010401.1 | *Acinetobacter baumannii* | p1ABAYE | 98 | 98 | 7.0E-32 | 8 |
| NC_010402.1 | *Acinetobacter baumannii* | p2ABAYE | 96 | 100 | 8.0E-41 | 8 |
| NC_010394.1 | *Mycobacterium abscessus* | unnamed | 95 | 100 | 2.0E-38 | 13 |
| NC_010396.1 | *Acinetobacter baumannii* | p2ABSDF | 97 | 100 | 3.0E-43 | 5 |
| NC_010398.1 | *Acinetobacter baumannii* | p3ABSDF | 95 | 100 | 2.0E-38 | 18 |
| NC_010404.1 | *Acinetobacter baumannii* | p3ABAYE | 95 | 100 | 2.0E-38 | 591 |
| NC_010466.1 | *Leuconostoc citreum* | pLCK2 | 97 | 100 | 3.0E-43 | 2 |
| NC_010470.1 | *Leuconostoc citreum* | pLCK1 | 98 | 100 | 1.0E-45 | 2 |
| NC_010488.1 | *Escherichia coli* | pSMS35_130 | 96 | 90 | 7.0E-35 | 16 |
| NC_010510.1 | *Methylobacterium radiotolerans* | pMRAD01 | 95 | 100 | 2.0E-38 | 1093 |
| NC_010481.1 | *Acinetobacter baumannii* | pABIR | 96 | 97 | 5.0E-39 | 12 |
| NC_010500.1 | *Salmonella enterica* | pUO-SbR5 | 100 | 94 | 8.0E-47 | 2 |
| NC_003227.1 | *Corynebacterium glutamicum* | pTET3 | 96 | 100 | 8.0E-41 | 5 |
| NC_003292.1 | *Escherichia coli* | R46 | 99 | 94 | 2.0E-44 | 2 |
| NC_003296.1 | *Ralstonia solanacearum* | pGMI1000MP | 95 | 100 | 2.0E-38 | 1821 |
| NC_003350.1 | *Pseudomonas putida* | pWW0 | 95 | 100 | 2.0E-38 | 130 |
| NC_010603.1 | *Lactobacillus reuteri* | pLR581 | 98 | 95 | 1.0E-42 | 2 |
| NC_010605.1 | *Acinetobacter baumannii* | pACICU1 | 95 | 100 | 2.0E-38 | 97 |
| NC_010606.1 | *Acinetobacter baumannii* | pACICU2 | 95 | 100 | 2.0E-38 | 139 |
| NC_003384.1 | *Salmonella enterica* | pHCM1 | 96 | 100 | 8.0E-41 | 4 |
| NC_010540.1 | *Lactococcus garvieae* | pKL0018 | 100 | 93 | 3.0E-46 | 1 |
| NC_010558.1 | *Escherichia coli* | pIP1206 | 99 | 95 | 5.0E-45 | 5 |
| NC_010726.1 | *Klebsiella pneumoniae* | pMET-1 | 100 | 94 | 8.0E-47 | 1 |
| NC_010813.1 | *Corynebacterium aurimucosum* | pET44827 | 97 | 100 | 3.0E-43 | 1 |
| NC_010937.1 | *Clostridium perfringens* | pCW3 | 95 | 100 | 2.0E-38 | 6 |
| NC_010941.1 | *Actinobacillus pleuropneumoniae* | ABB7_B | 96 | 99 | 1.0E-27 | 2 |
| NC_010841.1 | *Agrobacterium rhizogenes* | pRi2659 | 96 | 92 | 5.0E-36 | 1 |
| NC_010848.1 | *Flavobacterium* sp. | pOAD2 | 96 | 100 | 8.0E-41 | 5 |
| NC_010870.1 | *Klebsiella pneumoniae* | pK29 | 96 | 100 | 8.0E-41 | 5 |
| NC_010919.1 | *Aeromonas hydrophila* | pRA3 | 96 | 100 | 8.0E-41 | 6 |
| NC_010935.1 | *Comamonas testosteroni* | pCNB | 95 | 100 | 2.0E-38 | 802 |
| NC_010891.1 | *Pseudomonas* sp. | pCT14 | 95 | 100 | 2.0E-26 | 7 |
| NC_010886.1 | *Klebsiella pneumoniae* | pK245 | 96 | 100 | 8.0E-41 | 4 |
| NC_010980.1 | *Enterococcus faecium* | pVEF3 | 100 | 100 | 2.0E-50 | 4 |
| NC_010996.1 | *Rhizobium etli* | pB | 96 | 100 | 8.0E-41 | 4 |
| NC_010997.1 | *Rhizobium etli* | pC | 95 | 100 | 2.0E-38 | 2 |
| NC_010998.1 | *Rhizobium etli* | pA | 96 | 92 | 5.0E-36 | 1 |
| NC_003430.1 | *Uncultured bacterium in activated sludge* | pB4 | 96 | 94 | 3.0E-37 | 10 |
| NC_011077.1 | *Salmonella enterica* | pCVM29188_101 | 95 | 100 | 2.0E-38 | 5 |
| NC_011092.1 | *Salmonella enterica* | pCVM19633_110 | 97 | 92 | 2.0E-38 | 12 |
| NC_011143.1 | *Phenylobacterium zucineum* | unnamed | 97 | 90 | 3.0E-37 | 1 |
| NC_003486.1 | *Klebsiella pneumoniae* | pJHCMW1 | 100 | 94 | 8.0E-47 | 1 |
| NC_011339.1 | *Bacillus cereus* | pH308197_258 | 100 | 100 | 2.0E-50 | 2 |
| NC_011352.1 | *Lactobacillus casei* | plca36 | 97 | 101 | 7.0E-29 | 1 |
| NC_011368.1 | *Rhizobium leguminosarum* | pRLG201 | 95 | 100 | 2.0E-38 | 4 |
| NC_011370.1 | *Rhizobium leguminosarum* | pRLG203 | 96 | 92 | 5.0E-36 | 1 |
| NC_011371.1 | *Rhizobium leguminosarum* | pRLG204 | 99 | 97 | 3.0E-46 | 1 |
| NC_011385.1 | *Klebsiella pneumoniae* | plasmid 12 | 96 | 100 | 8.0E-41 | 5 |
| NC_011419.1 | *Escherichia coli* | pSE11-1 | 98 | 99 | 5.0E-45 | 2 |
| NC_011602.1 | *Escherichia coli* | pE2348-2 | 96 | 93 | 1.0E-36 | 5 |
| NC_011617.1 | *Klebsiella pneumoniae* | pKP96 | 96 | 100 | 8.0E-41 | 5 |
| NC_011642.1 | *Enterococcus faecalis* | pMG2200 | 98 | 94 | 5.0E-42 | 1 |
| NC_011667.1 | *Thauera* sp. | pTha01 | 95 | 100 | 2.0E-38 | 14 |
| NC_011752.1 | *Escherichia coli* | 55989p | 98 | 97 | 8.0E-44 | 1 |
| NC_011749.1 | *Escherichia coli* | p1ESCUM | 98 | 100 | 1.0E-45 | 4 |
| NC_011892.1 | *Methylobacterium nodulans* | pMNOD01 | 95 | 100 | 2.0E-38 | 6 |
| NC_011964.1 | *Escherichia coli* | pAPEC-O103-ColBM | 99 | 101 | 3.0E-46 | 3 |
| NC_011961.1 | *Thermomicrobium roseum* | unnamed | 95 | 100 | 2.0E-38 | 91 |
| NC_011982.1 | *Agrobacterium vitis* | pTiS4 | 96 | 97 | 5.0E-39 | 1 |
| NC_011984.1 | *Agrobacterium vitis* | pAtS4c | 95 | 100 | 2.0E-38 | 1 |
| NC_012520.1 | *Rhodococcus opacus* | pROB01 | 96 | 96 | 2.0E-38 | 1 |
| NC_012527.1 | *Deinococcus deserti* | plasmid 1 | 95 | 100 | 2.0E-38 | 228 |
| NC_012555.1 | *Enterobacter cloacae* | pEC-IMP | 96 | 100 | 8.0E-41 | 38 |
| NC_012556.1 | *Enterobacter cloacae* | pEC-IMPQ | 95 | 100 | 2.0E-38 | 253 |
| NC_012586.1 | *Sinorhizobium fredii* | pNGR234b | 95 | 100 | 2.0E-38 | 18 |
| NC_012690.1 | *Escherichia coli* | peH4H | 100 | 94 | 8.0E-47 | 3 |
| NC_012692.1 | *Escherichia coli* | pAR060302 | 100 | 94 | 8.0E-47 | 3 |
| NC_012693.1 | *Salmonella enterica* | pAM04528 | 100 | 100 | 2.0E-50 | 1 |
| NC_012780.1 | *Eubacterium eligens* | unnamed | 97 | 100 | 3.0E-43 | 8 |
| NC_012811.1 | *Methylobacterium extorquens* | megaplasmid | 95 | 100 | 2.0E-38 | 19 |
| NC_012813.1 | *Acinetobacter baumannii* | pABVA01 | 96 | 90 | 2.0E-32 | 2 |
| NC_012848.1 | *Rhizobium leguminosarum* | pR132501 | 95 | 100 | 2.0E-38 | 4 |
| NC_012849.1 | *Ralstonia pickettii* | pRp12D02 | 95 | 100 | 2.0E-38 | 79 |
| NC_012855.1 | *Ralstonia pickettii* | pRp12D01 | 97 | 96 | 8.0E-41 | 1 |
| NC_012858.1 | *Rhizobium leguminosarum* | pR132502 | 96 | 92 | 5.0E-36 | 2 |
| NC_012885.1 | *Aeromonas hydrophila* | pRA1 | 97 | 100 | 3.0E-43 | 1 |
| NC_012923.1 | *Streptococcus suis BM407* | pBM407 | 95 | 100 | 2.0E-38 | 8 |
| NC_012987.1 | *Methylobacterium extorquens* | p1METDI | 96 | 90 | 7.0E-35 | 1 |
| NC_013056.1 | *Acinetobacter calcoaceticus* | pMMCU1 | 100 | 100 | 2.0E-50 | 2 |
| NC_000914.2 | *Sinorhizobium fredii* | pNGR234a | 97 | 94 | 3.0E-37 | 3 |
| NC_013122.1 | *Escherichia coli* | pEK499 | 96 | 100 | 8.0E-41 | 5 |
| NC_013176.1 | *Pseudomonas putida* | pW2 | 95 | 100 | 2.0E-26 | 52 |
| NC_013190.1 | *Candidatus Accumulibacter phosphatis* | pAph02 | 96 | 95 | 8.0E-38 | 6 |
| NC_013191.1 | *Candidatus Accumulibacter phosphatis* | pAph03 | 96 | 100 | 8.0E-41 | 5 |
| NC_013193.1 | *Candidatus Accumulibacter phosphatis* | pAph01 | 96 | 95 | 8.0E-38 | 8 |
| NC_010847.2 | *Paracoccus aminophilus* | pAMI2 | 95 | 100 | 2.0E-38 | 4 |
| NC_013277.1 | *Acinetobacter baumannii* | pMMA2 | 97 | 100 | 3.0E-43 | 3 |
| NC_013356.1 | *Zymomonas mobilis* | pZA1001 | 96 | 93 | 1.0E-36 | 2 |
| NC_013365.1 | *Escherichia coli* | pO111_1 | 96 | 100 | 8.0E-41 | 9 |
| NC_013437.1 | *Salmonella enterica* | pSLT-BT | 100 | 94 | 8.0E-47 | 2 |
| NC_013506.1 | *Acinetobacter baumannii* | pMMCU2 | 95 | 100 | 2.0E-38 | 11 |
| NC_013545.1 | *Sinorhizobium meliloti* | pSmeSM11a | 96 | 90 | 7.0E-35 | 1 |
| NC_013666.1 | *Burkholderia cepacia* | pIJB1 | 95 | 100 | 2.0E-26 | 72 |
| NC_004566.1 | *Lactobacillus fermentum* | pLME300 | 100 | 100 | 2.0E-50 | 1 |
| NC_013773.1 | *Uncultured bacterium in activated sludge* | pGNB2 | 98 | 100 | 1.0E-45 | 1 |
| NC_004574.1 | *Ruegeria sp.* | pSD25 | 95 | 100 | 2.0E-38 | 80 |
| NC_013780.1 | *Aeromonas hydrophila* | pAH3680 | 97 | 90 | 3.0E-37 | 2 |
| NC_013855.1 | *Azospirillum* sp. | pAB510a | 95 | 100 | 2.0E-38 | 54 |
| NC_013856.1 | *Azospirillum* sp. | pAB510b | 95 | 100 | 2.0E-38 | 42 |
| NC_013857.1 | *Azospirillum* sp. | pAB510c | 95 | 100 | 2.0E-38 | 57 |
| NC_013858.1 | *Azospirillum* sp. | pAB510d | 97 | 92 | 2.0E-38 | 1 |
| NC_013859.1 | *Azospirillum* sp. | pAB510e | 95 | 100 | 2.0E-38 | 436 |
| NC_013860.1 | *Azospirillum* sp. | pAB510f | 95 | 100 | 2.0E-38 | 1 |
| NC_013951.1 | *Klebsiella pneumoniae* | pKF3-140 | 96 | 100 | 8.0E-41 | 5 |
| NC_007972.2 | *Cupriavidus metallidurans* | pMOL28 | 95 | 100 | 2.0E-26 | 80 |
| NC_007974.2 | *Cupriavidus metallidurans* | megaplasmid | 95 | 100 | 2.0E-38 | 553 |
| NC_013963.1 | *Bacillus* sp. | pBS-01 | 97 | 90 | 3.0E-37 | 4 |
| NC_013970.1 | *Sphingomonas* sp. | pISP3 | 95 | 100 | 2.0E-38 | 11 |
| NC_014005.1 | *Sphingobium japonicum* | pUT1 | 96 | 100 | 8.0E-41 | 7 |
| NC_014007.1 | *Sphingobium japonicum* | pCHQ1 | 95 | 100 | 2.0E-38 | 32 |
| NC_014016.1 | *Klebsiella pneumoniae* | pKpQIL | 98 | 100 | 1.0E-45 | 1 |
| NC_004604.2 | *Bacillus megaterium* | pBM400 | 95 | 100 | 2.0E-38 | 646 |
| NC_014035.1 | *Rhodobacter capsulatus* | pRCB133 | 95 | 100 | 2.0E-38 | 16 |
| NC_014107.1 | *Enterobacter cloacae* | pECL_A | 95 | 100 | 2.0E-38 | 25 |
| NC_014105.1 | *Neisseria gonorrhoeae* | pEP5289 | 96 | 100 | 8.0E-41 | 2 |
| NC_014124.1 | *Pseudomonas putida* | pDK1 | 100 | 100 | 2.0E-50 | 1 |
| NC_014134.1 | *Leuconostoc kimchii* | LkipL4726 | 99 | 99 | 2.0E-47 | 1 |
| NC_014154.1 | *Thiomonas intermedia* | pTINT01 | 96 | 97 | 5.0E-39 | 5 |
| NC_014155.1 | *Thiomonas intermedia* | pTINT02 | 95 | 100 | 2.0E-38 | 159 |
| NC_014167.1 | *Corynebacterium resistens* | pJA144188 | 96 | 95 | 8.0E-38 | 36 |
| NC_014211.1 | *Nocardiopsis dassonvillei* | pNDAS01 | 95 | 100 | 2.0E-38 | 1163 |
| NC_014208.1 | *Klebsiella oxytoca* | pKOX105 | 96 | 100 | 8.0E-41 | 121 |
| NC_014231.1 | *Escherichia coli* | pKC394 | 96 | 100 | 8.0E-41 | 6 |
| NC_014309.1 | *Ralstonia solanacearum* | RCFBPv3_mp | 95 | 100 | 2.0E-38 | 5 |
| NC_014312.1 | *Klebsiella pneumoniae* | pKP048 | 97 | 96 | 8.0E-41 | 4 |
| NC_014368.1 | *Klebsiella pneumoniae* | pNL194 | 96 | 100 | 8.0E-41 | 6 |
| NC_004771.1 | *Pasteurella multocida* | pJR1 | 95 | 100 | 2.0E-38 | 2 |
| NC_004772.1 | *Pasteurella multocida* | pJR2 | 100 | 94 | 8.0E-47 | 1 |
| NC_014475.1 | *Enterococcus faecalis* | pWZ1668 | 100 | 100 | 2.0E-50 | 1 |
| NC_014478.1 | *Klebsiella pneumoniae* | unnamed | 98 | 90 | 1.0E-39 | 2 |
| NC_014476.1 | *Salmonella enterica* | pYT1 | 100 | 94 | 8.0E-47 | 2 |
| NC_014615.1 | *Escherichia coli* | pETN48 | 96 | 100 | 8.0E-41 | 6 |
| NC_014621.1 | *Ketogulonicigenium vulgare* | pYP1 | 95 | 100 | 2.0E-38 | 1121 |
| NC_014633.1 | *Ilyobacter polytropus* | pILYOP01 | 95 | 100 | 2.0E-38 | 214 |
| NC_014641.1 | *Achromobacter xylosoxidans* | pA81 | 95 | 100 | 2.0E-38 | 2513 |
| NC_014642.1 | *Achromobacter xylosoxidans* | pA82 | 96 | 90 | 7.0E-35 | 1 |
| NC_014557.1 | *Bacillus* sp. | pBS-02 | 100 | 100 | 2.0E-50 | 1 |
| NC_014725.1 | *Edwardsiella tarda* | pCK41 | 100 | 96 | 5.0E-48 | 1 |
| NC_014801.1 | *Campylobacter jejuni* | pTet | 95 | 100 | 2.0E-38 | 4 |
| NC_014811.1 | *Mycobacterium* sp. | pMSPYR101 | 95 | 100 | 2.0E-38 | 68 |
| NC_014843.1 | *Escherichia coli* | p3521 | 95 | 100 | 2.0E-38 | 2 |
| NC_014832.1 | *Paracoccus aminophilus* | pAMI7 | 96 | 94 | 3.0E-37 | 1 |
| NC_004840.1 | *Uncultured bacterium in activated sludge* | pB10 | 95 | 100 | 2.0E-26 | 67 |
| NC_014908.1 | *Alicycliphilus denitrificans* | pALIDE01 | 95 | 100 | 2.0E-38 | 90 |
| NC_014911.1 | *Alicycliphilus denitrificans* | pALIDE02 | 95 | 100 | 2.0E-38 | 498 |
| NC_014918.1 | *Mesorhizobium ciceri* | pMESCI01 | 96 | 90 | 7.0E-35 | 1 |
| NC_014959.1 | *Enterococcus faecium* | pS177 | 100 | 100 | 2.0E-50 | 1 |
| NC_015053.1 | *Bifidobacterium longum* | p157F-NC1 | 96 | 99 | 3.0E-40 | 5 |
| NC_015154.1 | *Klebsiella pneumoniae* | pc15-k | 98 | 100 | 1.0E-45 | 3 |
| NC_004966.1 | *Lactococcus lactis* | pAH82 | 100 | 95 | 2.0E-47 | 2 |
| NC_004944.1 | *Lactobacillus plantarum* | pMD5057 | 99 | 96 | 1.0E-45 | 2 |
| NC_004945.1 | *Corynebacterium glutamicum* | pCG4 | 96 | 100 | 8.0E-41 | 6 |
| NC_004956.1 | *Pseudomonas* sp. | pADP-1 | 95 | 100 | 2.0E-26 | 104 |
| NC_004954.1 | *Micrococcus* sp. | pSD10 | 96 | 97 | 5.0E-39 | 2 |
| NC_004998.1 | *Escherichia coli* | p1658/97 | 100 | 94 | 8.0E-47 | 2 |
| NC_005026.1 | *Bacteroides fragilis* | pBI143 | 97 | 100 | 3.0E-43 | 1 |
| NC_005023.1 | *Acidithiobacillus ferrooxidans* | pTF5 | 96 | 98 | 1.0E-39 | 2 |
| NC_005000.1 | *Enterococcus faecium* | pRUM | 100 | 100 | 2.0E-50 | 1 |
| NC_015169.1 | *Deinococcus proteolyticus* | pDEIPR01 | 96 | 93 | 1.0E-36 | 1 |
| NC_005088.1 | *Delftia acidovorans* | pUO1 | 95 | 100 | 2.0E-26 | 81 |
| NC_005206.1 | *Arcanobacterium pyogenes* | pAP2 | 96 | 100 | 8.0E-41 | 4 |
| NC_005211.1 | *Serratia marcescens* | R478 | 96 | 100 | 8.0E-41 | 3 |
| NC_005241.1 | *Cupriavidus necator* | pHG1 | 95 | 100 | 2.0E-38 | 18 |
| NC_005307.1 | *Gordonia westfalica* | pKB1 | 97 | 94 | 4.0E-30 | 3 |
| NC_005244.2 | *Pseudomonas* sp. | pND6-1 | 96 | 100 | 8.0E-41 | 2 |
| NC_005793.1 | *Achromobacter denitrificans* | pEST4011 | 95 | 100 | 2.0E-38 | 91 |
| NC_005912.1 | *Ralstonia eutropha* | pJP4 | 95 | 100 | 2.0E-26 | 75 |
| NC_005909.1 | *Pseudomonas alcaligenes* | pRA2 | 98 | 97 | 8.0E-44 | 1 |
| NC_006143.1 | *Aeromonas punctata* | pFBAOT6 | 96 | 100 | 8.0E-41 | 29 |
| NC_006352.1 | *Uncultured bacterium in activated sludge* | pTB11 | 97 | 91 | 8.0E-38 | 56 |
| NC_006362.1 | *Nocardia farcinica* | pNF1 | 96 | 91 | 2.0E-35 | 2 |
| NC_004464.2 | Citrobacter freundii | pCTX-M3 | 100 | 96 | 5.0E-48 | 1 |
| NC_006385.1 | Uncultured bacterium in activated sludge | pRSB101 | 96 | 100 | 8.0E-41 | 22 |
| NC_006388.1 | Uncultured bacterium in activated sludge | pB3 | 96 | 100 | 8.0E-41 | 8 |
| NC_006525.1 | Cupriavidus metallidurans | pMOL28 | 95 | 100 | 2.0E-38 | 73 |
| NC_006529.1 | Lactobacillus salivarius | pSF118-20 | 100 | 98 | 3.0E-49 | 1 |
| NC_006569.1 | *Ruegeria pomeroyi* | megaplasmid | 95 | 100 | 2.0E-38 | 7 |
| NC_006625.1 | *Klebsiella pneumoniae* | pK2044 | 96 | 99 | 3.0E-40 | 9 |
| NC_006663.1 | *Staphylococcus epidermidis* | pSERP | 100 | 100 | 2.0E-50 | 2 |
| NC_006671.1 | *Escherichia coli* | pAPEC-O2-R | 100 | 96 | 5.0E-48 | 1 |
| NC_006816.1 | *Salmonella enterica* | pU302L | 96 | 100 | 8.0E-41 | 4 |
| NC_006823.1 | *Azoarcus* sp. | plasmid 1 | 95 | 100 | 2.0E-38 | 55 |
| NC_006824.1 | *Azoarcus* sp. | plasmid 2 | 96 | 93 | 1.0E-36 | 10 |
| NC_006830.1 | *Achromobacter xylosoxidans* | pA81 | 96 | 95 | 8.0E-38 | 64 |
| NC_006856.1 | *Salmonella enterica* | pSC138 | 99 | 100 | 5.0E-48 | 3 |
| NC_006877.1 | *Acinetobacter baumannii* | pMAC | 98 | 100 | 1.0E-45 | 5 |
| NC_007100.1 | *Pseudomonas aeruginosa* | Rms149 | 97 | 99 | 1.0E-42 | 9 |
| NC_007336.1 | *Ralstonia eutropha* | megaplasmid | 96 | 98 | 1.0E-39 | 4 |
| NC_007337.1 | *Ralstonia eutropha* | plasmid 1 | 95 | 100 | 2.0E-26 | 77 |
| NC_007486.1 | *Rhodococcus erythropolis* | pREC1 | 96 | 100 | 8.0E-41 | 16 |
| NC_007502.1 | *Uncultured bacterium in river sediments* | pB8 | 96 | 98 | 1.0E-39 | 15 |
| NC_007675.1 | *Escherichia coli* | pAPEC-O2-ColV | 98 | 100 | 1.0E-45 | 1 |
| NC_007680.1 | *Uncultured bacterium in river sediments* | pTP6 | 100 | 100 | 2.0E-50 | 1 |
| NC_007763.1 | *Rhizobium etli* | p42b | 96 | 93 | 1.0E-36 | 1 |
| NC_007765.1 | *Rhizobium etli* | p42e | 95 | 100 | 2.0E-38 | 2 |
| NC_007766.1 | *Rhizobium etli* | p42f | 96 | 91 | 2.0E-35 | 1 |
| NC_007772.1 | *Clostridium perfringens* | pCPF4969 | 100 | 100 | 2.0E-50 | 1 |
| NC_007353.2 | *Sphingomonas* sp. | pA1 | 96 | 95 | 8.0E-38 | 5 |
| NC_007930.1 | *Lactobacillus salivarius* | pMP118 | 98 | 94 | 5.0E-42 | 4 |
| NC_007950.1 | *Polaromonas* sp. | plasmid 2 | 97 | 94 | 1.0E-39 | 1 |
| NC_007959.1 | *Nitrobacter hamburgensis* | plasmid 1 | 96 | 96 | 2.0E-38 | 2 |
| NC_002134.1 | *Escherichia coli* | R100 | 100 | 94 | 8.0E-47 | 2 |
| NC_008036.1 | *Sphingopyxis alaskensis* | unamed | 95 | 100 | 2.0E-38 | 3 |
| NC_008042.1 | *Ruegeria* sp. | unnamed | 95 | 100 | 2.0E-38 | 71 |
| NC_008043.1 | *Ruegeria* sp. | megaplasmid | 95 | 100 | 2.0E-38 | 668 |
