# Supplementary material for: Plasmid Metagenome Reveals High Levels of Antibiotic Resistance Genes and Mobile Genetic Elements in Activated Sludge
Source: PLoS One. 2011 Oct 10;6(10):e26041. doi: 10.1371/journal.pone.0026041 (PMC3189950; doi:10.1371/journal.pone.0026041)
Supplement: Table S6 — Matched high-throughput sequencing reads of antibiotic resistance genes (ARGs) in the activated sludge against Antibiotic Resistance Database (ARDB). (DOC) [file pone.0026041.s006.doc]

| No | ARDB accession number | Identity (%) ≥ | Hit length (AA) ≥ | E value ≤ | Number of reads | ARG name | Function | Related antibiotics |
| --- | --- | --- | --- | --- | --- | --- | --- | --- |
| 1 | AAG05347 | 91 | 33 | 1.0E-11 | 4 | [*bacA*](http://ardb.cbcb.umd.edu/cgi/ssquery.cgi?db=T&gn=baca) | Undecaprenyl pyrophosphate phosphatase | [bacitracin](http://ardb.cbcb.umd.edu/cgi/search.cgi?db=B&field=ab&term=bacitracin) |
| 2 | AAO38916 | 100 | 33 | 7.0E-13 | 1 | [*tetO*](http://ardb.cbcb.umd.edu/cgi/ssquery.cgi?db=T&gn=teto) | Ribosomal protection protein | [tetracycline](http://ardb.cbcb.umd.edu/cgi/search.cgi?db=B&field=ab&term=tetracycline) |
| 3 | AAQ92181 | 97 | 32 | 2.0E-11 | 14 | *mexB* | Multidrug resistance efflux pump | aminoglycoside, β-lactam, fluoroquinolone, tetracycline, tigecycline |
| 4 | AAR21617 | 100 | 33 | 3.0E-13 | 3 | [*sulI*](http://ardb.cbcb.umd.edu/cgi/ssquery.cgi?db=T&gn=sul1) | Sulfonamide-resistant dihydropteroate synthase | [sulfonamide](http://ardb.cbcb.umd.edu/cgi/search.cgi?db=B&field=ab&term=sulfonamide) |
| 5 | AAS48620 | 100 | 31 | 3.0E-13 | 1 | *bla_VEB-3_* | Class A β-lactamase. | [cephalosporin, penicillin](http://ardb.cbcb.umd.edu/cgi/search.cgi?db=B&field=ab&term=cephalosporin) |
| 6 | AAS92348 | 100 | 28 | 2.0E-10 | 20 | [*bla_OXA-10_*](http://ardb.cbcb.umd.edu/cgi/ssquery.cgi?db=T&gn=bl2d_oxa10) | Class D β-lactamase. | [cloxacillin, penicillin](http://ardb.cbcb.umd.edu/cgi/search.cgi?db=B&field=ab&term=cloxacillin) |
| 7 | AAW66497 | 100 | 30 | 4.0E-11 | 8 | *tet39* | Tetracycline efflux pump. | tetracycline |
| 8 | ABD46539 | 100 | 33 | 2.0E-14 | 1 | [*tetM*](http://ardb.cbcb.umd.edu/cgi/ssquery.cgi?db=T&gn=tetm) | Ribosomal protection protein | [tetracycline](http://ardb.cbcb.umd.edu/cgi/search.cgi?db=B&field=ab&term=tetracycline) |
| 9 | ABG36700 | 93 | 29 | 3.0E-09 | 7 | *sulI* | Sulfonamide-resistant dihydropteroate synthase | [sulfonamide](http://ardb.cbcb.umd.edu/cgi/search.cgi?db=B&field=ab&term=sulfonamide) |
| 10 | ABI81214 | 100 | 31 | 8.0E-12 | 9 | [*tetM*](http://ardb.cbcb.umd.edu/cgi/ssquery.cgi?db=T&gn=tetm) | Ribosomal protection protein | [tetracycline](http://ardb.cbcb.umd.edu/cgi/search.cgi?db=B&field=ab&term=tetracycline) |
| 11 | ABN11268 | 100 | 30 | 3.0E-12 | 1 | [*tetM*](http://ardb.cbcb.umd.edu/cgi/ssquery.cgi?db=T&gn=tetm) | Ribosomal protection protein | [tetracycline](http://ardb.cbcb.umd.edu/cgi/search.cgi?db=B&field=ab&term=tetracycline) |
| 12 | ABN80430 | 100 | 32 | 3.0E-12 | 1 | *bla_VEB-5_* | Class A β-lactamase. | [cephalosporin, penicillin](http://ardb.cbcb.umd.edu/cgi/search.cgi?db=B&field=ab&term=cephalosporin) |
| 13 | ABO42050 | 100 | 33 | 1.0E-12 | 2 | *aadB* | Aminoglycoside O-nucleotidylyltransferase, | dibekacin, gentamicin, kanamycin, sisomicin, tobramycin |
| 14 | ABP92086 | 100 | 33 | 3.0E-13 | *26* | [*tetM*](http://ardb.cbcb.umd.edu/cgi/ssquery.cgi?db=T&gn=tetm) | Ribosomal protection protein | [tetracycline](http://ardb.cbcb.umd.edu/cgi/search.cgi?db=B&field=ab&term=tetracycline) |
| 15 | ABQ96629 | 100 | 32 | 5.0E-12 | 11 | *sulI* | Sulfonamide-resistant dihydropteroate synthase | [sulfonamide](http://ardb.cbcb.umd.edu/cgi/search.cgi?db=B&field=ab&term=sulfonamide) |
| 16 | ABW72062 | 97 | 33 | 5.0E-12 | 8 | [*acrB*](http://ardb.cbcb.umd.edu/cgi/ssquery.cgi?db=T&gn=acrb) | Multidrug resistance efflux pump | acriflavin, aminoglycoside, β-lactam, glycylcycline, macrolide |
| 17 | ABZ01843 | 100 | 33 | 2.0E-14 | 35 | [*tetG*](http://ardb.cbcb.umd.edu/cgi/ssquery.cgi?db=T&gn=tetg) | Tetracycline efflux pump. | [tetracycline](http://ardb.cbcb.umd.edu/cgi/search.cgi?db=B&field=ab&term=tetracycline) |
| 18 | ACA23195 | 90 | 30 | 8.0E-10 | 2 | [*tetW*](http://ardb.cbcb.umd.edu/cgi/ssquery.cgi?db=T&gn=tetw) | Ribosomal protection protein | [tetracycline](http://ardb.cbcb.umd.edu/cgi/search.cgi?db=B&field=ab&term=tetracycline) |
| 19 | ACB20261 | 100 | 33 | 2.0E-12 | *1* | [*strA*](http://ardb.cbcb.umd.edu/cgi/ssquery.cgi?db=T&gn=aph6id) | Aminoglycoside O-phosphotransferase | [streptomycin](http://ardb.cbcb.umd.edu/cgi/search.cgi?db=B&field=ab&term=streptomycin) |
| 20 | ACJ05688 | 100 | 33 | 2.0E-13 | 13 | *ampC* | Class C β-lactamase | cephalosporin |
| 21 | BAB71966 | 91 | 33 | 6.0E-13 | 4 | [*tetA(P)*](http://ardb.cbcb.umd.edu/cgi/ssquery.cgi?db=T&gn=tetpa) | Tetracycline efflux pump | [tetracycline](http://ardb.cbcb.umd.edu/cgi/search.cgi?db=B&field=ab&term=tetracycline) |
| 22 | BAC77251 | 91 | 32 | 3.0E-10 | 15 | [*acrB*](http://ardb.cbcb.umd.edu/cgi/ssquery.cgi?db=T&gn=acrb) | Multidrug resistance efflux pump | acriflavin, aminoglycoside, β-lactam, glycylcycline, macrolide |
| 23 | CAC86407 | 97 | 32 | 3.0E-13 | 2 | [*strA*](http://ardb.cbcb.umd.edu/cgi/ssquery.cgi?db=T&gn=aph6id) | Aminoglycoside O-phosphotransferase | [streptomycin](http://ardb.cbcb.umd.edu/cgi/search.cgi?db=B&field=ab&term=streptomycin) |
| 24 | CAA06639 | 100 | 31 | 8.0E-12 | 9 | *ampC* | Class C β-lactamase | cephalosporin |
| 25 | CAD55718 | 97 | 31 | 7.0E-11 | 2 | [*tet36*](http://ardb.cbcb.umd.edu/cgi/ssquery.cgi?db=T&gn=tet36) | Ribosomal protection protein | [tetracycline](http://ardb.cbcb.umd.edu/cgi/search.cgi?db=B&field=ab&term=tetracycline) |
| 26 | CAE50480 | 100 | 31 | 7.0E-13 | 7 | *sulI* | Sulfonamide-resistant dihydropteroate synthase | [sulfonamide](http://ardb.cbcb.umd.edu/cgi/search.cgi?db=B&field=ab&term=sulfonamide) |
| 27 | CAE53425 | 100 | 33 | 1.0E-12 | 5 | [*sulII*](http://ardb.cbcb.umd.edu/cgi/ssquery.cgi?db=T&gn=sul2) | Sulfonamide-resistant dihydropteroate synthase | [sulfonamide](http://ardb.cbcb.umd.edu/cgi/search.cgi?db=B&field=ab&term=sulfonamide) |
| 28 | CAG74079 | 91 | 32 | 2.0E-09 | 11 | [*acrB*](http://ardb.cbcb.umd.edu/cgi/ssquery.cgi?db=T&gn=acrb) | Multidrug resistance efflux pump | acriflavin, aminoglycoside, β-lactam, glycylcycline, macrolide |
| 29 | CAI43343 | 100 | 27 | 6.0E-10 | 1 | [*cmlA*](http://ardb.cbcb.umd.edu/cgi/ssquery.cgi?db=T&gn=cml_e1) | Major facilitator superfamily transporter, chloramphenicol efflux pump. | [chloramphenicol](http://ardb.cbcb.umd.edu/cgi/search.cgi?db=B&field=ab&term=chloramphenicol) |
| 30 | CAJ00297 | 100 | 33 | 7.0E-13 | 7 | [*tetM*](http://ardb.cbcb.umd.edu/cgi/ssquery.cgi?db=T&gn=tetm) | Ribosomal protection protein | [tetracycline](http://ardb.cbcb.umd.edu/cgi/search.cgi?db=B&field=ab&term=tetracycline) |
| 31 | CAJ77026 | 100 | 27 | 2.0E-11 | 1 | [*strB*](http://ardb.cbcb.umd.edu/cgi/ssquery.cgi?db=T&gn=aph6id) | Aminoglycoside O-phosphotransferase | [streptomycin](http://ardb.cbcb.umd.edu/cgi/search.cgi?db=B&field=ab&term=streptomycin) |
| 32 | CAM88409 | 100 | 32 | 1.0E-12 | 1 | *floR* | Chloramphenicol efflux pump. | [chloramphenicol](http://ardb.cbcb.umd.edu/cgi/search.cgi?db=B&field=ab&term=chloramphenicol) |
| 33 | CAM88415 | 100 | 31 | 4.0E-14 | 3 | [*strA*](http://ardb.cbcb.umd.edu/cgi/ssquery.cgi?db=T&gn=aph6id) | Aminoglycoside O-phosphotransferase | [streptomycin](http://ardb.cbcb.umd.edu/cgi/search.cgi?db=B&field=ab&term=streptomycin) |
| 34 | CAY51926 | 91 | 33 | 3.0E-10 | 9 | [*mexW*](http://ardb.cbcb.umd.edu/cgi/ssquery.cgi?db=T&gn=mexw) | Multidrug resistance efflux pump |  |
| 35 | EEQ96502 | 90 | 30 | 2.0E-10 | 12 | [*bacA*](http://ardb.cbcb.umd.edu/cgi/ssquery.cgi?db=T&gn=baca) | Undecaprenyl pyrophosphate phosphatase | [bacitracin](http://ardb.cbcb.umd.edu/cgi/search.cgi?db=B&field=ab&term=bacitracin) |
| 36 | JQ1201 | 91 | 33 | 3.0E-13 | 1 | [*cmlA*](http://ardb.cbcb.umd.edu/cgi/ssquery.cgi?db=T&gn=cml_e1) | Chloramphenicol efflux pump. | [chloramphenicol](http://ardb.cbcb.umd.edu/cgi/search.cgi?db=B&field=ab&term=chloramphenicol) |
| 37 | NP_348076 | 93 | 30 | 3.0E-10 | 64 | *tetB(P)* | Ribosomal protection protein | [tetracycline](http://ardb.cbcb.umd.edu/cgi/search.cgi?db=B&field=ab&term=tetracycline) |
| 40 | NP_940742 | 93 | 28 | 6.0E-10 | 1 | [*tet33*](http://ardb.cbcb.umd.edu/cgi/ssquery.cgi?db=T&gn=tet33) | Tetracycline efflux pump. | [tetracycline](http://ardb.cbcb.umd.edu/cgi/search.cgi?db=B&field=ab&term=tetracycline) |
| 41 | Q82Y49 | 94 | 32 | 2.0E-11 | 2 | *bacA* | Undecaprenyl pyrophosphate phosphatase | [bacitracin](http://ardb.cbcb.umd.edu/cgi/search.cgi?db=B&field=ab&term=bacitracin) |
| 44 | YP_001144149 | 100 | 27 | 8.0E-12 | 1 | [*catB*](http://ardb.cbcb.umd.edu/cgi/ssquery.cgi?db=T&gn=cata13)*3* | Group A chloramphenicol acetyltransferase | [chloramphenicol](http://ardb.cbcb.umd.edu/cgi/search.cgi?db=B&field=ab&term=chloramphenicol) |
| 45 | YP_001186705 | 90 | 31 | 1.0E-10 | 5 | [*mexW*](http://ardb.cbcb.umd.edu/cgi/ssquery.cgi?db=T&gn=mexw) | Multidrug resistance efflux pump |  |
| 46 | YP_001350280 | 94 | 31 | 2.0E-09 | 11 | [*mexW*](http://ardb.cbcb.umd.edu/cgi/ssquery.cgi?db=T&gn=mexw) | Multidrug resistance efflux pump |  |
| 47 | YP_001416709 | 91 | 33 | 8.0E-12 | 7 | [*bacA*](http://ardb.cbcb.umd.edu/cgi/ssquery.cgi?db=T&gn=baca) | Undecaprenyl pyrophosphate phosphatase | [bacitracin](http://ardb.cbcb.umd.edu/cgi/search.cgi?db=B&field=ab&term=bacitracin) |
| 48 | YP_001563294 | 93 | 28 | 6.0E-09 | 9 | [*bacA*](http://ardb.cbcb.umd.edu/cgi/ssquery.cgi?db=T&gn=baca) | Undecaprenyl pyrophosphate phosphatase | [bacitracin](http://ardb.cbcb.umd.edu/cgi/search.cgi?db=B&field=ab&term=bacitracin) |
| 49 | YP_001571041 | 93 | 30 | 2.0E-09 | 175 | *macB* | Macrolide-specific efflux system | Macrolide |
| 50 | YP_001749316 | 91 | 33 | 1.0E-12 | 2 | [*mexF*](http://ardb.cbcb.umd.edu/cgi/ssquery.cgi?db=T&gn=mexf) | Multidrug resistance efflux pump | [chloramphenicol, fluoroquinolone](http://ardb.cbcb.umd.edu/cgi/search.cgi?db=B&field=ab&term=chloramphenicol) |
| 51 | YP_001836040 | 91 | 32 | 5.0E-11 | 4 | [*ermB*](http://ardb.cbcb.umd.edu/cgi/ssquery.cgi?db=T&gn=ermb) | rRNA adenine N-6-methyltransferase | lincosamide, macrolide, streptogramin_b |
| 52 | YP_001844878 | 91 | 33 | 7.0E-14 | 3 | *aadA* | Aminoglycoside O-phosphotransferase | gentamincin_b, kanamycin, lividomycin, neomycin, paromomycin, ribostamycin |
| 53 | YP_001908403 | 91 | 33 | 1.0E-11 | 11 | *acrB* | Multidrug resistance efflux pump | acriflavin, aminoglycoside, β-lactam, glycylcycline, macrolide |
| 54 | YP_001969930 | 90 | 30 | 2.0E-09 | 2 | *sulII* | Sulfonamide-resistant dihydropteroate synthase | [sulfonamide](http://ardb.cbcb.umd.edu/cgi/search.cgi?db=B&field=ab&term=sulfonamide) |
| 55 | YP_002029849 | 94 | 32 | 3.0E-10 | 21 | [*smeE*](http://ardb.cbcb.umd.edu/cgi/ssquery.cgi?db=T&gn=smee) | Multidrug resistance efflux pump. | [fluoroquinolone](http://ardb.cbcb.umd.edu/cgi/search.cgi?db=B&field=ab&term=fluoroquinolone) |
| 56 | YP_002030219 | 97 | 33 | 6.0E-12 | 9 | *smeB* | Multidrug resistance efflux pump. | [fluoroquinolone](http://ardb.cbcb.umd.edu/cgi/search.cgi?db=B&field=ab&term=fluoroquinolone) |
| 57 | YP_002112964 | 100 | 28 | 2.0E-11 | 10 | *aphA-2* | Aminoglycoside O-phosphotransferase | [streptomycin](http://ardb.cbcb.umd.edu/cgi/search.cgi?db=B&field=ab&term=streptomycin) |
| 58 | YP_002847505 | 94 | 33 | 9.0E-11 | 7 | [*mdtF*](http://ardb.cbcb.umd.edu/cgi/ssquery.cgi?db=T&gn=mdtf) | Multidrug resistance efflux pump | [doxorubicin, erythromycin](http://ardb.cbcb.umd.edu/cgi/search.cgi?db=B&field=ab&term=doxorubicin) |
| 59 | YP_002850805 | 100 | 29 | 2.0E-10 | 10 | *tetB(P)* | Ribosomal protection protein | [tetracycline](http://ardb.cbcb.umd.edu/cgi/search.cgi?db=B&field=ab&term=tetracycline) |
| 60 | YP_002890644 | 100 | 33 | 1.0E-12 | 15 | [*bacA*](http://ardb.cbcb.umd.edu/cgi/ssquery.cgi?db=T&gn=baca) | Undecaprenyl pyrophosphate phosphatase | [bacitracin](http://ardb.cbcb.umd.edu/cgi/search.cgi?db=B&field=ab&term=bacitracin) |
| 61 | YP_002894485 | 96 | 25 | 3.0E-08 | 6 | *aadA* | Aminoglycoside O-nucleotidylyltransferase | spectinomycin, streptomycin |
| 62 | YP_151445 | 94 | 32 | 1.0E-10 | 2 | *acrB* | Multidrug resistance efflux pump | acriflavin, aminoglycoside, β-lactam, glycylcycline, macrolide |
| 63 | YP_454364 | 91 | 33 | 5.0E-11 | 6 | *acrB* | Multidrug resistance efflux pump | acriflavin, aminoglycoside, β-lactam, glycylcycline, macrolide |
| 64 | YP_746446 | 100 | 31 | 1.0E-11 | 2 | [*bacA*](http://ardb.cbcb.umd.edu/cgi/ssquery.cgi?db=T&gn=baca) | Undecaprenyl pyrophosphate phosphatase | [bacitracin](http://ardb.cbcb.umd.edu/cgi/search.cgi?db=B&field=ab&term=bacitracin) |
| 65 | YP_932216 | 94 | 33 | 1.0E-11 | 14 | [*bacA*](http://ardb.cbcb.umd.edu/cgi/ssquery.cgi?db=T&gn=baca) | Undecaprenyl pyrophosphate phosphatase | [bacitracin](http://ardb.cbcb.umd.edu/cgi/search.cgi?db=B&field=ab&term=bacitracin) |
| 66 | YP_997055 | 91 | 32 | 9.0E-11 | 1 | [*bacA*](http://ardb.cbcb.umd.edu/cgi/ssquery.cgi?db=T&gn=baca) | Undecaprenyl pyrophosphate phosphatase | [bacitracin](http://ardb.cbcb.umd.edu/cgi/search.cgi?db=B&field=ab&term=bacitracin) |
| 67 | ZP_01363330 | 94 | 31 | 1.0E-10 | 15 | *mexB* | Multidrug resistance efflux pump | aminoglycoside, β-lactam, fluoroquinolone, tetracycline, tigecycline |
| 68 | ZP_02632674 | 97 | 33 | 2.0E-12 | 4 | [*tetB*](http://ardb.cbcb.umd.edu/cgi/ssquery.cgi?db=T&gn=tetpb)*(P)* | Ribosomal protection protein | [tetracycline](http://ardb.cbcb.umd.edu/cgi/search.cgi?db=B&field=ab&term=tetracycline) |
| 69 | ZP_03075977 | 94 | 33 | 7.0E-13 | 1 | [*arnA*](http://ardb.cbcb.umd.edu/cgi/ssquery.cgi?db=T&gn=arna) | Antimicrobial peptides. | [polymyxin](http://ardb.cbcb.umd.edu/cgi/search.cgi?db=B&field=ab&term=polymyxin) |
| 70 | ZP_03222019 | 94 | 33 | 1.0E-11 | 12 | *acrB* | Multidrug resistance efflux pump | acriflavin, aminoglycoside, β-lactam, glycylcycline, macrolide |
| 71 | ZP_03486481 | 97 | 32 | 2.0E-12 | 2 | [*tet40*](http://ardb.cbcb.umd.edu/cgi/ssquery.cgi?db=T&gn=tet40) | Tetracycline efflux pump | [tetracycline](http://ardb.cbcb.umd.edu/cgi/search.cgi?db=B&field=ab&term=tetracycline) |
| 72 | ZP_03552050 | 94 | 32 | 1.0E-11 | 3 | [*bacA*](http://ardb.cbcb.umd.edu/cgi/ssquery.cgi?db=T&gn=baca) | Undecaprenyl pyrophosphate phosphatase | [bacitracin](http://ardb.cbcb.umd.edu/cgi/search.cgi?db=B&field=ab&term=bacitracin) |
| 73 | ZP_03823621 | 90 | 31 | 2.0E-11 | 1 | [*adeA*](http://ardb.cbcb.umd.edu/cgi/ssquery.cgi?db=T&gn=adea) | Multidrug resistance efflux pump | [aminoglycoside, fluoramphenicol](http://ardb.cbcb.umd.edu/cgi/search.cgi?db=B&field=ab&term=aminoglycoside) |
| 74 | ZP_04435342 | 100 | 33 | 1.0E-12 | 13 | [*tetM*](http://ardb.cbcb.umd.edu/cgi/ssquery.cgi?db=T&gn=tetm) | Ribosomal protection protein | [tetracycline](http://ardb.cbcb.umd.edu/cgi/search.cgi?db=B&field=ab&term=tetracycline) |
| 75 | ZP_04543532 | 93 | 27 | 1.0E-09 | 1 | [*bla_CFX-a_*](http://ardb.cbcb.umd.edu/cgi/ssquery.cgi?db=T&gn=bl2e_cfxa) | Class A β-lactamase. | [cephalosporin](http://ardb.cbcb.umd.edu/cgi/search.cgi?db=B&field=ab&term=cephalosporin) |
| 76 | ZP_04615616 | 91 | 32 | 2.0E-10 | 3 | *acrB* | Multidrug resistance efflux pump | acriflavin, aminoglycoside, β-lactam, glycylcycline, macrolide |
| 77 | ZP_04620400 | 91 | 32 | 4.0E-11 | 2 | *acrB* | Multidrug resistance efflux pump | acriflavin, aminoglycoside, β-lactam, glycylcycline, macrolide |
| 78 | ZP_04635098 | 90 | 31 | 3.0E-10 | 6 | *acrB* | Multidrug resistance efflux pump | acriflavin, aminoglycoside, β-lactam, glycylcycline, macrolide |
