# Supplementary material for: Plasmid Metagenome Reveals High Levels of Antibiotic Resistance Genes and Mobile Genetic Elements in Activated Sludge
Source: PLoS One. 2011 Oct 10;6(10):e26041. doi: 10.1371/journal.pone.0026041 (PMC3189950; doi:10.1371/journal.pone.0026041)
Supplement: Table S7 — Matched high-throughput sequencing reads of integrons and gene cassettes in the activated sludge of Shatin STP. (DOC) [file pone.0026041.s007.doc]

| Microbial species | Integron | gene cassette | Accession number | Identity (%) ≥ | Hit length (bp) ≥ | E value ≤ | Number of reads |
| --- | --- | --- | --- | --- | --- | --- | --- |
| *Acinetobacter baumannii* | *intI1* | *ampC* | AY228469 | 90.22 | 92 | 1.00E-25 | 126 |
| *Corynebacterium diphtheriae* | *intI1* |  | BX248359 | 91.04 | 67 | 6.00E-18 | 55 |
| *Enterobacter cloacae* | *intI1* | *bla_VEB-3_* | AY536519 | 100 | 94 | 2.00E-48 | 1 |
| *Pseudomonas aeruginosa* | *intI1* | *bla_VIM-2_*, *catB8, aacA4*, *aadA2*, *qacEΔ1* | AF418284 | 94.57 | 92 | 3.00E-35 | 4 |
| *Pseudomonas aeruginosa* | *intI1* | *aadA2b*, *sulI* | D43625 | 100 | 91 | 1.00E-46 | 1 |
| *Pseudomonas stutzeri* | *intI1* |  | EF648213 | 90 | 70 | 2.00E-17 | 39 |
| *Roseiflexus castenholzii* | *intI1* |  | NC_009767 | 90.91 | 77 | 2.00E-21 | 1 |
| *Salmonella enterica* | *intI1* | *dfrA1*, *aadA1, qacEΔ1*, *sulI* | AY524415 | 98.53 | 68 | 2.00E-30 | 5 |
| *Salmonella enterica* | *intI1* | *aadA2*, *linG* | DQ836009 | 93 | 100 | 3.00E-35 | 24 |
| *Serratia marcescens* | *intI3* | *bla_IMP-1_*, *aacA4* | D50438 | 95.7 | 93 | 3.00E-38 | 2 |
| *Thauera* sp. | *intI1* |  | EU327991 | 97.94 | 97 | 2.00E-45 | 1 |
| *Yersinia pestis* | *intI1* | *aadA2*, *sulI* | CP000603 | 92.59 | 54 | 2.00E-17 | 5 |
| Uncultured bacterium | New integrase gene | *PiGC15.HVF18* | AB290253 | 90.7 | 86 | 2.00E-24 | 1 |
| Uncultured bacterium | New integrase gene | *MuGC6.Sym6* | AB290259 | 90 | 50 | 3.00E-10 | 1 |
| Uncultured bacterium | New integrase gene | *SuezGC30.1* | AB547007 | 90.36 | 83 | 1.00E-22 | 1 |
| Uncultured bacterium | New integrase gene | *SuezGC57.1* | AB547034 | 90.7 | 86 | 2.00E-24 | 1 |
| Uncultured bacterium | New integrase gene | *NWAB_1_9_1_1* | AM912948 | 91.07 | 56 | 9.00E-14 | 2 |
| uncultured bacterium | *intI1* |  | AY283624 | 92.19 | 64 | 2.00E-18 | 1 |
| uncultured bacterium | *intI1* | *qacEΔ2* | FJ172393 | 97.3 | 74 | 1.00E-31 | 1 |
| uncultured bacterium | *intI1* | *aadA24* | FJ377586 | 100 | 95 | 6.00E-49 | 1 |
| uncultured bacterium | *intI1* |  | FJ377607 | 91.07 | 56 | 9.00E-14 | 1 |
| uncultured bacterium | *intI1* |  | FJ377612 | 90.91 | 77 | 2.00E-21 | 1 |
| uncultured bacterium | *intI1* | *qacEΔ1* | FJ663011 | 94.51 | 91 | 1.00E-34 | 13 |
| uncultured bacterium | *intI1* | *qacEΔ1*, *qacF* | FJ820123 | 100 | 100 | 6.00E-52 | 12 |
| uncultured bacterium | *intI1* | *qacH* | FJ820150 | 91.07 | 56 | 9.00E-14 | 1 |
| uncultured bacterium | *intI1* |  | FM866490 | 90.77 | 65 | 9.00E-17 | 17 |
| uncultured bacterium | *intI1* |  | FM867352 | 90.63 | 64 | 4.00E-16 | 3 |
| uncultured bacterium | *intI2* |  | GQ161094 | 100 | 100 | 6.00E-52 | 1 |
| uncultured bacterium | *intI2* |  | DQ282331 | 94.12 | 51 | 1.00E-15 | 12 |
| uncultured bacterium | *intI2* |  | DQ282364 | 90 | 50 | 3.00E-10 | 3 |
| uncultured bacterium | *intI2* |  | FJ615794 | 100 | 100 | 6.00E-52 | 1 |
