# Supplementary material for: Plasmid Metagenome Reveals High Levels of Antibiotic Resistance Genes and Mobile Genetic Elements in Activated Sludge
Source: PLoS One. 2011 Oct 10;6(10):e26041. doi: 10.1371/journal.pone.0026041 (PMC3189950; doi:10.1371/journal.pone.0026041)
Supplement: Table S8 — Matched high-throughput sequencing reads of insertion sequence common region transposases in plasmid metagenome of activated sludge of Shatin STP. (DOC) [file pone.0026041.s008.doc]

| No. | Transposon | Identity (%) ≥ | Hit length (bp) ≥ | E value ≤ | Number of reads | Bacterial hosts |
| --- | --- | --- | --- | --- | --- | --- |
| 1 | ISAav1 | 90 | 60 | 2.0E-14 | 168 | *Acidovorax avenae* |
| 2 | ISAav3 | 90 | 50 | 9.0E-11 | 70 |
| 3 | ISAba1 | 90 | 92 | 3.0E-26 | 175 | [*Acinetobacter baumannii*](http://www.ncbi.nlm.nih.gov/Taxonomy/Browser/wwwtax.cgi?name=Acinetobacter+baumannii) |
| 4 | ISAba125 | 93 | 57 | 3.0E-17 | 155 |
| 5 | ISAba2 | 90 | 100 | 1.0E-28 | 198 |
| 6 | ISAba3 | 91 | 98 | 8.0E-30 | 306 |
| 7 | ISAba7 | 90 | 70 | 7.0E-18 | 84 |
| 8 | ISAca1 | 93 | 75 | 1.0E-25 | 1 | *Acinetobacter calcoaceticus* |
| 9 | ISAfe7 | 90 | 50 | 9.0E-11 | 6 | [*Acidithiobacillus ferrooxidans*](http://www.ncbi.nlm.nih.gov/Taxonomy/Browser/wwwtax.cgi?name=Acidithiobacillus+ferrooxidans) |
| 10 | ISAni1 | 92 | 50 | 4.0E-13 | 2 | *Corynebacterium striatum* plasmid pTP10 |
| 11 | ISAs1 | 99 | 90 | 4.0E-44 | 2 | *Aeromonas salmonicida* |
| 12 | ISAs2 | 97 | 100 | 2.0E-45 | 2 |
| 13 | ISAzo1 | 92 | 50 | 4.0E-13 | 2 | [*Azoarcus* sp*.*](http://www.ncbi.nlm.nih.gov/Taxonomy/Browser/wwwtax.cgi?name=Azoarcus+sp.) plasmid 2 |
| 14 | ISAzo3 | 90 | 50 | 9.0E-11 | 5 |
| 15 | ISAzo4 | 90 | 90 | 5.0E-25 | 13 |
| 16 | ISAzo6 | 90 | 50 | 9.0E-11 | 43 |
| 17 | ISBcen15 | 92 | 61 | 3.0E-17 | 1 | [*Burkholderia cenocepacia*](http://www.ncbi.nlm.nih.gov/Taxonomy/Browser/wwwtax.cgi?name=Burkholderia+cenocepacia) |
| 18 | ISBcen18 | 90 | 83 | 3.0E-23 | 8 |
| 19 | ISBcen3 | 90 | 50 | 9.0E-11 | 2 | [*Burkholderia cenocepacia*](http://www.ncbi.nlm.nih.gov/Taxonomy/Browser/wwwtax.cgi?name=Burkholderia+cenocepacia) |
| 20 | ISBj1 | 92 | 53 | 6.0E-15 | 01 | *Bradyrhizobium japonicum* |
| 21 | ISBli1 | 90 | 50 | 9.0E-11 | 3 | *Brevibacterium linens* |
| 22 | ISBli2 | 91 | 64 | 1.0E-16 | 1 |
| 23 | ISBlo1 | 91 | 58 | 2.0E-15 | 1 | [*Bifidobacterium longum*](http://www.ncbi.nlm.nih.gov/Taxonomy/Browser/wwwtax.cgi?name=Bifidobacterium+longum) |
| 24 | ISBlo2 | 90 | 73 | 1.0E-19 | 3 |
| 25 | ISBlo3 | 95 | 84 | 2.0E-33 | 3 |
| 26 | ISBlo4 | 90 | 70 | 7.0E-18 | 66 |
| 27 | ISBlo5 | 95 | 100 | 1.0E-40 | 1 |
| 28 | ISBlo7 | 93 | 57 | 3.0E-17 | 1 |
| 29 | ISBlo8 | 90 | 52 | 6.0E-12 | 1 |
| 30 | ISBmu2 | 90 | 52 | 6.0E-12 | 2 | *Burkholderia multivorans* |
| 31 | ISBp1 | 91 | 57 | 6.0E-15 | 1 | [*Burkholderia pseudomallei*](http://www.ncbi.nlm.nih.gov/Taxonomy/Browser/wwwtax.cgi?name=Burkholderia+pseudomallei) |
| 32 | ISBrsp1 | 92 | 52 | 2.0E-14 | 1 | [*Bradyrhizobium* sp.](http://www.ncbi.nlm.nih.gov/Taxonomy/Browser/wwwtax.cgi?name=Bradyrhizobium+sp.) |
| 33 | ISBthe1 | 98 | 81 | 2.0E-36 | 1 | [*Bacteroides thetaiotaomicron*](http://www.ncbi.nlm.nih.gov/Taxonomy/Browser/wwwtax.cgi?name=Bacteroides+thetaiotaomicron) |
| 34 | ISBuce1 | 90 | 51 | 2.0E-11 | 8 | [*Burkholderia cepacia*](http://www.ncbi.nlm.nih.gov/Taxonomy/Browser/wwwtax.cgi?name=Burkholderia+cepacia) |
| 35 | ISC1041 | 96 | 100 | 6.0E-43 | 27 | *Sulfolobus solfataricus* |
| 36 | ISCc3 | 90 | 70 | 7.0E-18 | 66 | [*Caulobacter crescentus*](http://www.ncbi.nlm.nih.gov/Taxonomy/Browser/wwwtax.cgi?name=Caulobacter+crescentus) |
| 37 | ISCmi2 | 90 | 52 | 6.0E-12 | 1 | [*Clavibacter michiganensis*](http://www.ncbi.nlm.nih.gov/Taxonomy/Browser/wwwtax.cgi?name=Clavibacter+michiganensis) |
| 38 | ISCpe4 | 91 | 90 | 2.0E-27 | 2 | [*Clostridium perfringens*](http://www.ncbi.nlm.nih.gov/Taxonomy/Browser/wwwtax.cgi?name=Clostridium+perfringens) |
| 39 | ISCpe5 | 97 | 62 | 5.0E-25 | 1 |
| 40 | ISDar2 | 93 | 55 | 4.0E-16 | 1 | [*Dechloromonas aromatica*](http://www.ncbi.nlm.nih.gov/Taxonomy/Browser/wwwtax.cgi?name=Dechloromonas+aromatica) |
| 41 | ISEc1 | 95 | 96 | 3.0E-38 | 2 | [*Escherichia coli*](http://www.ncbi.nlm.nih.gov/Taxonomy/Browser/wwwtax.cgi?name=Escherichia+coli) |
| 42 | ISEc9 | 91 | 92 | 1.0E-28 | 8 | [*Escherichia coli*](http://www.ncbi.nlm.nih.gov/Taxonomy/Browser/wwwtax.cgi?name=Escherichia+coli)plasmid pST01 |
| 43 | ISEcp1 | 100 | 100 | 2.0E-52 | 3 |
| 44 | ISEfm1 | 94 | 51 | 4.0E-16 | 93 | [*Enterococcus faecium*](http://www.ncbi.nlm.nih.gov/Taxonomy/Browser/wwwtax.cgi?name=Enterococcus+faecium) |
| 45 | ISFlsp1 | 91 | 64 | 2.0E-14 | 3 | [*Flavobacterium* sp](http://www.ncbi.nlm.nih.gov/Taxonomy/Browser/wwwtax.cgi?name=Flavobacterium+sp)*.* |
| 46 | ISFsp3 | 90 | 50 | 9.0E-11 | 54 | [*Frankia* sp*.*](http://www.ncbi.nlm.nih.gov/Taxonomy/Browser/wwwtax.cgi?name=Frankia+sp.) |
| 47 | ISGsu4 | 90 | 51 | 2.0E-11 | 1 | [*Geobacter sulfurreducens*](http://www.ncbi.nlm.nih.gov/Taxonomy/Browser/wwwtax.cgi?name=Geobacter+sulfurreducens) |
| 48 | ISHne1 | 90 | 63 | 4.0E-16 | 11 | [*Hyphomonas neptunium*](http://www.ncbi.nlm.nih.gov/Taxonomy/Browser/wwwtax.cgi?name=Hyphomonas+neptunium) |
| 49 | ISKpn3 | 98 | 100 | 1.0E-47 | 1 | *Klebsiella pneumoniae* plasmid pRDDHA |
| 50 | ISL6 | 93 | 89 | 1.0E-31 | 2 | [*Lactobacillus delbrueckii*](http://www.ncbi.nlm.nih.gov/Taxonomy/Browser/wwwtax.cgi?name=Lactobacillus+delbrueckii) |
| 51 | ISLxx3 | 90 | 80 | 2.0E-21 | 17 | [*Leifsonia xyli*](http://www.ncbi.nlm.nih.gov/Taxonomy/Browser/wwwtax.cgi?name=Leifsonia+xyli) |
| 52 | ISLxx4 | 92 | 53 | 6.0E-15 | 1 |
| 53 | ISMav1 | 90 | 51 | 2.0E-11 | 4 | [*Mycobacterium avium*](http://www.ncbi.nlm.nih.gov/Taxonomy/Browser/wwwtax.cgi?name=Mycobacterium+avium) |
| 54 | ISMav2 | 91 | 64 | 1.0E-16 | 2 |
| 55 | ISMca1 | 94 | 63 | 7.0E-21 | 4 | [*Methylococcus capsulatus*](http://www.ncbi.nlm.nih.gov/Taxonomy/Browser/wwwtax.cgi?name=Methylococcus+capsulatus) |
| 56 | ISMca2 | 92 | 89 | 3.0E-29 | 6 |
| 57 | ISMca3 | 90 | 50 | 9.0E-11 | 8 |
| 58 | ISMca5 | 90 | 50 | 9.0E-11 | 40 |
| 59 | ISMca7 | 90 | 50 | 9.0E-11 | 11 |
| 60 | ISMmg1 | 92 | 59 | 4.0E-16 | 1 | [*Magnetospirillum magnetotacticum*](http://www.ncbi.nlm.nih.gov/Taxonomy/Browser/wwwtax.cgi?name=Magnetospirillum+magnetotacticum) |
| 61 | ISMpa1 | 90 | 60 | 2.0E-14 | 6 | [*Mycobacterium avium*](http://www.ncbi.nlm.nih.gov/Taxonomy/Browser/wwwtax.cgi?name=Mycobacterium+avium) |
| 62 | ISMt1 | 90 | 50 | 9.0E-11 | 5 | [*Mycobacterium tuberculosis*](http://www.ncbi.nlm.nih.gov/Taxonomy/Browser/wwwtax.cgi?name=Mycobacterium+tuberculosis) |
| 63 | ISMt3 | 91 | 55 | 1.0E-13 | 2 |
| 64 | ISNGR8 | 90 | 70 | 7.0E-18 | 6 | [*Rhizobium* sp](http://www.ncbi.nlm.nih.gov/Taxonomy/Browser/wwwtax.cgi?name=Rhizobium+sp)*.* plasmid pNGR234a |
| 65 | ISNme1 | 92 | 52 | 2.0E-14 | 1 | [*Neisseria meningitidis*](http://www.ncbi.nlm.nih.gov/Taxonomy/Browser/wwwtax.cgi?name=Neisseria+meningitidis) |
| 66 | ISOur1 | 90 | 100 | 1.0E-28 | 22 | [*Oligella urethralis*](http://www.ncbi.nlm.nih.gov/Taxonomy/Browser/wwwtax.cgi?name=Oligella+urethralis) |
| 67 | ISPa14 | 90 | 94 | 2.0E-27 | 40 | [*Pseudomonas aeruginosa*](http://www.ncbi.nlm.nih.gov/Taxonomy/Browser/wwwtax.cgi?name=Pseudomonas+aeruginosa) |
| 68 | ISPa7 | 90 | 60 | 2.0E-14 | 1 | [*Pseudomonas aeruginosa*](http://www.ncbi.nlm.nih.gov/Taxonomy/Browser/wwwtax.cgi?name=Pseudomonas+aeruginosa) |
| 69 | ISPa8 | 93 | 54 | 2.0E-15 | 1 |
| 70 | ISPosp1 | 90 | 100 | 1.0E-28 | 4 | *Pseudomonas entomophila* |
| 71 | ISPp2 | 90 | 52 | 6.0E-12 | 1 | [*Pseudomonas putida*](http://www.ncbi.nlm.nih.gov/Taxonomy/Browser/wwwtax.cgi?name=Pseudomonas+putida) |
| 72 | ISPpa1 | 90 | 91 | 1.0E-25 | 12 | [*Paracoccus pantotrophus*](http://www.ncbi.nlm.nih.gov/Taxonomy/Browser/wwwtax.cgi?name=Paracoccus+pantotrophus) |
| 73 | ISPpa2 | 90 | 60 | 2.0E-14 | 28 |
| 74 | ISPpa3 | 90 | 81 | 4.0E-22 | 5 |
| 75 | ISPpa4 | 90 | 71 | 2.0E-18 | 6 |
| 76 | ISPpa5 | 91 | 99 | 2.0E-30 | 3 |
| 77 | ISPps1 | 90 | 80 | 2.0E-21 | 1048 | [*Pseudomonas huttiensis*](http://www.ncbi.nlm.nih.gov/Taxonomy/Browser/wwwtax.cgi?name=Pseudomonas+huttiensis) |
| 78 | ISPpu12 | 94 | 86 | 3.0E-32 | 8 | [*Pseudomonas putida*](http://www.ncbi.nlm.nih.gov/Taxonomy/Browser/wwwtax.cgi?name=Pseudomonas+putida) |
| 79 | ISPpu7 | 91 | 68 | 4.0E-19 | 1 |
| 80 | ISPre1 | 91 | 66 | 7.0E-18 | 3 | [*Pseudomonas resinovorans*](http://www.ncbi.nlm.nih.gov/Taxonomy/Browser/wwwtax.cgi?name=Pseudomonas+resinovorans) |
| 81 | ISPre3 | 90 | 50 | 9.0E-11 | 1 |
| 82 | ISPsp2 | 97 | 96 | 6.0E-43 | 3 | [*Pseudomonas* sp*.*](http://www.ncbi.nlm.nih.gov/Taxonomy/Browser/wwwtax.cgi?name=Pseudomonas+sp.)plasmid pEST1226 |
| 83 | ISPsp3 | 90 | 50 | 9.0E-11 | 15 |
| 84 | ISPst2 | 92 | 94 | 8.0E-30 | 12 | [*Pseudomonas stutzeri*](http://www.ncbi.nlm.nih.gov/Taxonomy/Browser/wwwtax.cgi?name=Pseudomonas+stutzeri) |
| 85 | ISPst3 | 90 | 50 | 9.0E-11 | 40 |
| 86 | ISPst4 | 91 | 75 | 7.0E-21 | 7 |
| 87 | ISPst5 | 91 | 53 | 2.0E-12 | 1 |
| 88 | ISPsy1 | 91 | 53 | 2.0E-12 | 2 | [*Pseudomonas syringae*](http://www.ncbi.nlm.nih.gov/Taxonomy/Browser/wwwtax.cgi?name=Pseudomonas+syringae) |
| 89 | ISPsy17 | 91 | 55 | 1.0E-13 | 1 |
| 90 | ISPsy20 | 90 | 50 | 9.0E-11 | 14 |
| 91 | ISPsy24 | 94 | 51 | 4.0E-16 | 1 |
| 92 | ISR1 | 90 | 60 | 2.0E-14 | 9 | [*Rhizobium lupini*](http://www.ncbi.nlm.nih.gov/Taxonomy/Browser/wwwtax.cgi?name=Rhizobium+lupini)plasmid pRP4 |
| 93 | ISRhsp1 | 90 | 52 | 6.0E-12 | 1 | [*Rhodobacter sphaeroides*](http://www.ncbi.nlm.nih.gov/Taxonomy/Browser/wwwtax.cgi?name=Rhodobacter+sphaeroides) |
| 94 | ISRl2 | 93 | 68 | 2.0E-21 | 1 | [*Rhizobium leguminosarum*](http://www.ncbi.nlm.nih.gov/Taxonomy/Browser/wwwtax.cgi?name=Rhizobium+leguminosarum) |
| 95 | ISRle39b | 91 | 65 | 3.0E-17 | 1 | [*Rhizobium leguminosarum*](http://www.ncbi.nlm.nih.gov/Taxonomy/Browser/wwwtax.cgi?name=Rhizobium+leguminosarum) |
| 96 | ISRm1 | 90 | 83 | 3.0E-23 | 2 | [*Rhizobium meliloti*](http://www.ncbi.nlm.nih.gov/Taxonomy/Browser/wwwtax.cgi?name=Rhizobium+meliloti) plasmid pRmeSU47a |
| 97 | ISRm13 | 90 | 50 | 9.0E-11 | 3 | [*Sinorhizobium meliloti*](http://www.ncbi.nlm.nih.gov/Taxonomy/Browser/wwwtax.cgi?name=Sinorhizobium+meliloti)plasmid pSym |
| 98 | ISRm14 | 90 | 91 | 1.0E-25 | 3 |
| 99 | ISRm3G | 90 | 50 | 9.0E-11 | 2 | [*Rhizobium meliloti*](http://www.ncbi.nlm.nih.gov/Taxonomy/Browser/wwwtax.cgi?name=Rhizobium+meliloti) plasmid pRmeGR4b |
| 100 | ISRm4-1 | 90 | 80 | 2.0E-21 | 5 | [*Rhizobium meliloti*](http://www.ncbi.nlm.nih.gov/Taxonomy/Browser/wwwtax.cgi?name=Rhizobium+meliloti) plasmid pRmNT4 |
| 101 | ISRm4-3 | 91 | 58 | 2.0E-15 | 1 | [*Sinorhizobium meliloti*](http://www.ncbi.nlm.nih.gov/Taxonomy/Browser/wwwtax.cgi?name=Sinorhizobium+meliloti) |
| 102 | ISRm5 | 91 | 82 | 5.0E-25 | 1 | [*Rhizobium meliloti*](http://www.ncbi.nlm.nih.gov/Taxonomy/Browser/wwwtax.cgi?name=Rhizobium+meliloti) |
| 103 | ISRm6 | 91 | 74 | 3.0E-20 | 2 | [*Rhizobium meliloti*](http://www.ncbi.nlm.nih.gov/Taxonomy/Browser/wwwtax.cgi?name=Rhizobium+meliloti) plasmid pRmeGR4 |
| 104 | ISRme4 | 90 | 50 | 9.0E-11 | 10 | [*Cupriavidus metallidurans*](http://www.ncbi.nlm.nih.gov/Taxonomy/Browser/wwwtax.cgi?name=Cupriavidus+metallidurans) |
| 105 | ISRo1 | 90 | 51 | 2.0E-11 | 4 | [*Rhodococcus opacus*](http://www.ncbi.nlm.nih.gov/Taxonomy/Browser/wwwtax.cgi?name=Rhodococcus+opacus)plasmid pHG201 |
| 106 | ISRso12 | 90 | 62 | 2.0E-15 | 2 | [*Ralstonia solanacearum*](http://www.ncbi.nlm.nih.gov/Taxonomy/Browser/wwwtax.cgi?name=Ralstonia+solanacearum) |
| 107 | ISRso14 | 91 | 58 | 2.0E-15 | 1 | [*Ralstonia solanacearum*](http://www.ncbi.nlm.nih.gov/Taxonomy/Browser/wwwtax.cgi?name=Ralstonia+solanacearum) |
| 108 | ISRso7 | 90 | 70 | 7.0E-18 | 7 | [*Ralstonia solanacearum*](http://www.ncbi.nlm.nih.gov/Taxonomy/Browser/wwwtax.cgi?name=Ralstonia+solanacearum) |
| 109 | ISRsp2 | 91 | 86 | 5.0E-25 | 2 | [Rhizobium sp.](http://www.ncbi.nlm.nih.gov/Taxonomy/Browser/wwwtax.cgi?name=Rhizobium+sp.) plasmid pNGR234a |
| 110 | ISRsp5 | 90 | 71 | 2.0E-18 | 1 | [*Rhizobium* sp.](http://www.ncbi.nlm.nih.gov/Taxonomy/Browser/wwwtax.cgi?name=Rhizobium+sp.) |
| 111 | ISRtr1 | 90 | 50 | 9.0E-11 | 4 | [*Rhizobium tropici*](http://www.ncbi.nlm.nih.gov/Taxonomy/Browser/wwwtax.cgi?name=Rhizobium+tropici) |
| 112 | ISS1T | 100 | 100 | 2.0E-52 | 1 | [*Lactococcus lactis*](http://www.ncbi.nlm.nih.gov/Taxonomy/Browser/wwwtax.cgi?name=Lactococcus+lactis)plasmid pSK08 |
| 113 | ISS1W | 99 | 100 | 4.0E-50 | 4 | [*Lactococcus lactis*](http://www.ncbi.nlm.nih.gov/Taxonomy/Browser/wwwtax.cgi?name=Lactococcus+lactis) plasmid pWV05 |
| 114 | ISS1Z | 99 | 100 | 4.0E-50 | 1 | [*Lactococcus lactis*](http://www.ncbi.nlm.nih.gov/Taxonomy/Browser/wwwtax.cgi?name=Lactococcus+lactis)plasmid pOZS550 |
| 115 | ISSag3 | 92 | 79 | 1.0E-25 | 20 | [*Streptococcus agalactiae*](http://www.ncbi.nlm.nih.gov/Taxonomy/Browser/wwwtax.cgi?name=Streptococcus+agalactiae) |
| 116 | ISSco1 | 91 | 75 | 7.0E-21 | 2 | [*Streptomyces coelicolor*](http://www.ncbi.nlm.nih.gov/Taxonomy/Browser/wwwtax.cgi?name=Streptomyces+coelicolor)cosmid J11 |
| 117 | ISSdy1 | 93 | 98 | 1.0E-34 | 2 | [*Streptococcus dysgalactiae*](http://www.ncbi.nlm.nih.gov/Taxonomy/Browser/wwwtax.cgi?name=Streptococcus+dysgalactiae)*subs* |
| 118 | ISSep1 | 92 | 59 | 4.0E-16 | 3 | [*Staphylococcus epidermidis*](http://www.ncbi.nlm.nih.gov/Taxonomy/Browser/wwwtax.cgi?name=Staphylococcus+epidermidis) |
| 119 | ISShsp1 | 91 | 66 | 7.0E-18 | 1 | [*Sphingomonas* sp.](http://www.ncbi.nlm.nih.gov/Taxonomy/Browser/wwwtax.cgi?name=Sphingomonas+sp.) |
| 120 | ISSm2 | 90 | 50 | 9.0E-11 | 679 | [*Serratia marcescens*](http://www.ncbi.nlm.nih.gov/Taxonomy/Browser/wwwtax.cgi?name=Serratia+marcescens) |
| 121 | ISSmi2 | 92 | 50 | 4.0E-13 | 1 | [*Streptococcus mitis*](http://www.ncbi.nlm.nih.gov/Taxonomy/Browser/wwwtax.cgi?name=Streptococcus+mitis) |
| 122 | ISSod1 | 91 | 55 | 1.0E-13 | 1 | [*Shewanella oneidensis*](http://www.ncbi.nlm.nih.gov/Taxonomy/Browser/wwwtax.cgi?name=Shewanella+oneidensis) |
| 123 | ISSod9 | 90 | 50 | 9.0E-11 | 19 |
| 124 | ISSp1 | 91 | 96 | 1.0E-28 | 15 | [*Sphingomonas paucimobilis*](http://www.ncbi.nlm.nih.gov/Taxonomy/Browser/wwwtax.cgi?name=Sphingomonas+paucimobilis) |
| 125 | ISSpo2 | 92 | 50 | 4.0E-13 | 1 | [*Silicibacter pomeroyi*](http://www.ncbi.nlm.nih.gov/Taxonomy/Browser/wwwtax.cgi?name=Silicibacter+pomeroyi) |
| 126 | ISSpo7 | 90 | 50 | 9.0E-11 | 18 |
| 127 | ISSpo9 | 90 | 91 | 1.0E-25 | 27 |
| 128 | ISSsp126 | 90 | 50 | 9.0E-11 | 1 | [*Sphingomonas* sp.](http://www.ncbi.nlm.nih.gov/Taxonomy/Browser/wwwtax.cgi?name=Sphingomonas+sp.) |
| 129 | ISSsu3 | 90 | 100 | 1.0E-28 | 15 | [*Streptococcus suis*](http://www.ncbi.nlm.nih.gov/Taxonomy/Browser/wwwtax.cgi?name=Streptococcus+suis) |
| 130 | ISSth1 | 90 | 50 | 9.0E-11 | 31 | [*Streptococcus thermophilus*](http://www.ncbi.nlm.nih.gov/Taxonomy/Browser/wwwtax.cgi?name=Streptococcus+thermophilus) |
| 131 | ISTesp1 | 90 | 60 | 2.0E-14 | 8 | [*Terrabacter* sp.](http://www.ncbi.nlm.nih.gov/Taxonomy/Browser/wwwtax.cgi?name=Terrabacter+sp.) |
| 132 | ISTesp3 | 90 | 60 | 2.0E-14 | 21 |
| 133 | ISUnCu3 | 90 | 52 | 6.0E-12 | 9 | [Plasmid QKH54](http://www.ncbi.nlm.nih.gov/Taxonomy/Browser/wwwtax.cgi?name=Plasmid+QKH54) |
| 134 | ISUnCu4 | 90 | 50 | 9.0E-11 | 2 |
| 135 | ISVme1 | 91 | 93 | 3.0E-29 | 2 | [*Vibrio metschnikovii*](http://www.ncbi.nlm.nih.gov/Taxonomy/Browser/wwwtax.cgi?name=Vibrio+metschnikovii) |
| 136 | ISVsa3 | 95 | 92 | 8.0E-36 | 9 | [*Vibrio salmonicida*](http://www.ncbi.nlm.nih.gov/Taxonomy/Browser/wwwtax.cgi?name=Vibrio+salmonicida) |
| 137 | ISVsp1 | 91 | 88 | 3.0E-26 | 1 | [*Verrucomicrobium spinosum*](http://www.ncbi.nlm.nih.gov/Taxonomy/Browser/wwwtax.cgi?name=Verrucomicrobium+spinosum) |
| 138 | ISVsp13 | 91 | 74 | 3.0E-20 | 3 |
| 139 | ISWz1 | 91 | 54 | 4.0E-13 | 3 | [*Weeksella zoohelcum*](http://www.ncbi.nlm.nih.gov/Taxonomy/Browser/wwwtax.cgi?name=Weeksella+zoohelcum) |
| 140 | ISXa1 | 92 | 51 | 1.0E-13 | 8 | [*Xanthobacter autotrophicus*](http://www.ncbi.nlm.nih.gov/Taxonomy/Browser/wwwtax.cgi?name=Xanthobacter+autotrophicus) |
| 141 | ISXac2 | 91 | 89 | 2.0E-24 | 4 | [*Xanthomonas axonopodis*](http://www.ncbi.nlm.nih.gov/Taxonomy/Browser/wwwtax.cgi?mode=Info&id=92831&lvl=3&lin=f&keep=1&srchmode=1&unlock) |
| 142 | ISXac3 | 91 | 53 | 2.0E-12 | 1 |
| 143 | ISXax1 | 90 | 60 | 6.0E-12 | 2 | [*Xanthomonas axonopodis*](http://www.ncbi.nlm.nih.gov/Taxonomy/Browser/wwwtax.cgi?name=Xanthomonas+axonopodis) |
| 144 | ISXc5 | 91 | 75 | 7.0E-21 | 4 | [*Xanthomonas campestris*](http://www.ncbi.nlm.nih.gov/Taxonomy/Browser/wwwtax.cgi?name=Xanthomonas+campestris)plasmid pXW45J |
| 145 | ISXo1 | 91 | 56 | 2.0E-14 | 9 | [*Xanthomonas oryzae*](http://www.ncbi.nlm.nih.gov/Taxonomy/Browser/wwwtax.cgi?name=Xanthomonas+oryzae) |
| 146 | ISXo7 | 90 | 80 | 2.0E-21 | 14 | [*Xanthomonas oryzae*](http://www.ncbi.nlm.nih.gov/Taxonomy/Browser/wwwtax.cgi?name=Xanthomonas+oryzae) |
| 147 | ISXo9 | 90 | 50 | 9.0E-11 | 1 | *Xanthomonas oryzae* |
| 148 | KIS | 92 | 50 | 4.0E-13 | 1 | [*Lactobacillus delbrueckii*](http://www.ncbi.nlm.nih.gov/Taxonomy/Browser/wwwtax.cgi?name=Lactobacillus+delbrueckii)bacteriophage LL-K |
