# Supplementary material for: Plasmid Metagenome Reveals High Levels of Antibiotic Resistance Genes and Mobile Genetic Elements in Activated Sludge
Source: PLoS One. 2011 Oct 10;6(10):e26041. doi: 10.1371/journal.pone.0026041 (PMC3189950; doi:10.1371/journal.pone.0026041)
Supplement: Table S9 — Matched high-throughput sequencing reads of virulence factors in plasmid metagenome of activated sludge of Shatin STP. (DOC) [file pone.0026041.s009.doc]

| No. | Virulence factors | Identity (%) ≥ | Hit length (bp) ≥ | E value ≤ | Number of reads | Bacterial hosts | Functions |
| --- | --- | --- | --- | --- | --- | --- | --- |
| 1 | VFG0032 | 93 | 54 | 3.0E-15 | 1 | *Bordetella pertussis* | probable sugar transferase |
| 2 | VFG0036 | 98 | 52 | 4.0E-21 | 1 |  | lipopolysaccharide biosynthesis protein |
| 3 | VFG0064 | 90 | 51 | 5.0E-11 | 1 |  | type III secretion pore protein |
| 4 | VFG0079 | 90 | 50 | 2.0E-10 | 2 | *Listeria monocytogenes* | endopeptidase |
| 5 | VFG0111 | 90 | 62 | 3.0E-15 | 5 | *Pseudomonas aeruginosa* | type 4 fimbrial precursor |
| 6 | VFG0112 | 90 | 50 | 2.0E-10 | 35 |  | type 4 fimbrial precursor |
| 7 | VFG0114 | 90 | 80 | 4.0E-21 | 3 |  | type 4 prepilin peptidase |
| 8 | VFG0116 | 90 | 51 | 5.0E-11 | 4 |  | two-component response regulator |
| 9 | VFG0120 | 90 | 61 | 1.0E-14 | 1 |  | sigma factor |
| 10 | VFG0121 | 90 | 50 | 2.0E-10 | 4 |  | alginate biosynthesis protein |
| 11 | VFG0122 | 90 | 60 | 5.0E-14 | 2 |  | GDP-mannose 6-dehydrogenase |
| 12 | VFG0127 | 90 | 51 | 5.0E-11 | 1 |  | outer membrane protein |
| 13 | VFG0130 | 90 | 50 | 2.0E-10 | 7 |  | alginate o-acetyltransferase |
| 14 | VFG0133 | 92 | 71 | 1.0E-20 | 3 |  | phosphomannose isomerase |
| 15 | VFG0160 | 90 | 50 | 2.0E-10 | 1 |  | pyoverdine biosynthesis protein |
| 16 | VFG0161 | 91 | 54 | 8.0E-13 | 3 |  | pyoverdine synthetase |
| 17 | VFG0165 | 90 | 50 | 2.0E-10 | 8 |  | pyochelin synthetase |
| 18 | VFG0182 | 90 | 60 | 5.0E-14 | 42 |  | general secretion pathway protein |
| 19 | VFG0232 | 90 | 60 | 5.0E-14 | 3 | *Neisseria meningitidis* | twitching motility protein |
| 20 | VFG0431 | 92 | 53 | 1.0E-14 | 1 | *Salmonella enterica* | polysaccharide biosynthesis protein |
| 21 | VFG0477 | 92 | 50 | 8.0E-13 | 2 |  | sigma S factor of RNA polymerase |
| 22 | VFG0524 | 92 | 51 | 2.0E-13 | 1 |  | lyase transcriptional activator |
| 23 | VFG0576 | 91 | 74 | 6.0E-20 | 1 |  | ssDNA-binding protein |
| 24 | VFG0601 | 93 | 84 | 3.0E-28 | 1 | *Shigella flexneri* | transposase |
| 25 | VFG0612 | 90 | 50 | 2.0E-10 | 2 |  | conserved hypothetical protein |
| 26 | VFG1026 | 91 | 98 | 2.0E-29 | 2 |  | streptomycin adenyltransferase |
| 27 | VFG1028 | 91 | 65 | 5.0E-17 | 17 |  | integrase |
| 28 | VFG1029 | 91 | 82 | 1.0E-24 | 4 |  | transposase |
| 29 | VFG1030 | 92 | 71 | 1.0E-20 | 8 |  | resolvase |
| 30 | VFG1031 | 90 | 80 | 4.0E-21 | 164 |  | transposase |
| 31 | VFG1066 | 90 | 51 | 5.0E-11 | 1 |  | unknown |
| 32 | VFG1102 | 90 | 52 | 1.0E-11 | 1 | *Vibrio cholerae* | DNA methylase |
| 33 | VFG1214 | 90 | 51 | 5.0E-11 | 4 | *Pseudomonas aeruginosa* | two-component response regulator |
| 34 | VFG1218 | 90 | 62 | 3.0E-15 | 1 |  | type 4 fimbrial biogenesis protein |
| 35 | VFG1223 | 90 | 60 | 5.0E-14 | 52 |  | twitching motility protein |
| 36 | VFG1224 | 90 | 50 | 2.0E-10 | 25 |  | twitching motility protein |
| 37 | VFG1225 | 90 | 52 | 1.0E-11 | 11 |  | twitching motility protein |
| 38 | VFG1226 | 92 | 62 | 1.0E-17 | 2 |  | twitching motility protein |
| 39 | VFG1228 | 90 | 50 | 2.0E-10 | 7 |  | twitching motility protein |
| 40 | VFG1231 | 90 | 52 | 1.0E-11 | 3 |  | chemotactic signal transduction system |
| 41 | VFG1240 | 92 | 62 | 1.0E-17 | 4 |  | flagellar basal-body rod protein FlgG |
| 42 | VFG1242 | 90 | 60 | 5.0E-14 | 3 |  | flagellar P-ring protein precursor FlgI |
| 43 | VFG1246 | 90 | 51 | 5.0E-11 | 2 |  | flagellin type B |
| 44 | VFG1248 | 90 | 50 | 2.0E-10 | 42 |  | transcriptional regulator FleQ |
| 45 | VFG1249 | 90 | 60 | 3.0E-09 | 4 |  | two-component response regulator |
| 46 | VFG1252 | 92 | 59 | 9.0E-16 | 1 |  | flagellar motor switch protein FliG |
| 47 | VFG1254 | 90 | 63 | 9.0E-16 | 5 |  | flagellum-specific ATP synthase FliI |
| 48 | VFG1259 | 90 | 50 | 2.0E-10 | 7 |  | flagellar biosynthetic protein FliP |
| 49 | VFG1263 | 91 | 97 | 6.0E-29 | 12 |  | flagellar biosynthesis protein FlhA |
| 50 | VFG1269 | 90 | 52 | 1.0E-11 | 1 | *Bordetella pertussis* | cyclolysin secretion ATP-binding protein |
| 51 | VFG1381 | 90 | 50 | 2.0E-10 | 51 | *Mycobacterium tuberculosis* | aceA |
| 52 | VFG1382 | 90 | 70 | 1.0E-17 | 2 |  | umaA2 |
| 53 | VFG1383 | 90 | 80 | 6.0E-20 | 20 |  | hypothetical protein |
| 54 | VFG1386 | 91 | 100 | 1.0E-30 | 5 |  | phoP |
| 55 | VFG1389 | 91 | 77 | 9.0E-22 | 5 |  | hypothetical protein |
| 56 | VFG1390 | 90 | 90 | 1.0E-24 | 21 |  | hypothetical protein |
| 57 | VFG1391 | 90 | 50 | 2.0E-10 | 36 |  | narG |
| 58 | VFG1392 | 90 | 100 | 3.0E-28 | 3 |  | sigE |
| 59 | VFG1396 | 90 | 50 | 2.0E-10 | 77 |  | katG |
| 60 | VFG1399 | 90 | 50 | 2.0E-10 | 20 |  | glnA1 |
| 61 | VFG1403 | 91 | 53 | 3.0E-12 | 1 |  | mbtB |
| 62 | VFG1405 | 90 | 50 | 2.0E-10 | 233 |  | sigA |
| 63 | VFG1406 | 90 | 51 | 5.0E-11 | 7 |  | ideR |
| 64 | VFG1411 | 90 | 50 | 2.0E-10 | 5 |  | leuD |
| 65 | VFG1412 | 90 | 50 | 2.0E-10 | 24 |  | sigH |
| 66 | VFG1413 | 91 | 56 | 5.0E-14 | 1 |  | sigF |
| 67 | VFG1416 | 90 | 80 | 4.0E-21 | 4 |  | panD |
| 68 | VFG1417 | 92 | 59 | 9.0E-16 | 1 |  | panC |
| 69 | VFG1419 | 91 | 56 | 5.0E-14 | 7 |  | fbpA |
| 70 | VFG1420 | 91 | 54 | 8.0E-13 | 2 |  | pirG |
| 71 | VFG1421 | 92 | 87 | 1.0E-27 | 1 |  | sodA |
| 72 | VFG1583 | 98 | 100 | 2.0E-47 | 2 | *Escherichia coli* | hypothetical protein |
| 73 | VFG1811 | 90 | 50 | 2.0E-10 | 5 | *Mycobacterium tuberculosis* | fbpC2 |
| 74 | VFG1812 | 90 | 73 | 2.0E-19 | 3 |  | fbpB |
| 75 | VFG1814 | 90 | 50 | 2.0E-10 | 85 |  | narH |
| 76 | VFG1815 | 91 | 68 | 9.0E-19 | 1 |  | narJ |
| 77 | VFG1816 | 90 | 51 | 5.0E-11 | 2 |  | narI |
| 78 | VFG1824 | 90 | 50 | 2.0E-10 | 5 |  | hypothetical protein |
| 79 | VFG1825 | 91 | 100 | 1.0E-30 | 4 |  | hypothetical protein |
| 80 | VFG1826 | 90 | 60 | 5.0E-14 | 21 |  | relA |
| 81 | VFG1880 | 90 | 50 | 2.0E-10 | 1 | *Legionella pneumophila* | pilus assembly protein PilB |
| 82 | VFG2012 | 90 | 62 | 3.0E-15 | 1 |  | flagellar biosynthetic protein FlhA |
| 83 | VFG2045 | 90 | 50 | 2.0E-10 | 4 | *Bordetella pertussis* | virulence sensor protein |
| 84 | VFG2059 | 91 | 55 | 2.0E-13 | 1 | *Pseudomonas aeruginosa* | ATP-binding component of ABC transporter |
| 85 | VFG2060 | 90 | 50 | 2.0E-10 | 2 |  | serine/threonine protein kinase PpkA |
| 86 | VFG2063 | 90 | 52 | 1.0E-11 | 4 |  | hypothetical protein |
| 87 | VFG2069 | 91 | 53 | 3.0E-12 | 3 |  | hypothetical protein |
| 88 | VFG2070 | 90 | 100 | 3.0E-28 | 17 |  | hypothetical protein |
| 89 | VFG2071 | 92 | 50 | 8.0E-13 | 1 |  | hypothetical protein |
| 90 | VFG2073 | 91 | 53 | 3.0E-12 | 1 |  | hypothetical protein |
| 91 | VFG2076 | 90 | 81 | 9.0E-22 | 24 |  | ClpA/B-type chaperone |
| 92 | VFG2077 | 91 | 64 | 2.0E-16 | 2 |  | hypothetical protein |
| 93 | VFG2219 | 91 | 56 | 5.0E-14 | 1 | *Brucella melitensis* | cyclic beta 1-2 glucan synthetase |
| 94 | VFG2220 | 91 | 66 | 1.0E-17 | 2 |  | Phosphoglucomutase pgm |
| 95 | VFG2401 | 92 | 52 | 5.0E-14 | 1 | *Mycobacterium tuberculosis* | hypothetical protein |
