# Supplementary figures and images for: Plasmid Metagenome Reveals High Levels of Antibiotic Resistance Genes and Mobile Genetic Elements in Activated Sludge
Source: PLoS One. 2011 Oct 10;6(10):e26041. doi: 10.1371/journal.pone.0026041 (PMC3189950; doi:10.1371/journal.pone.0026041)

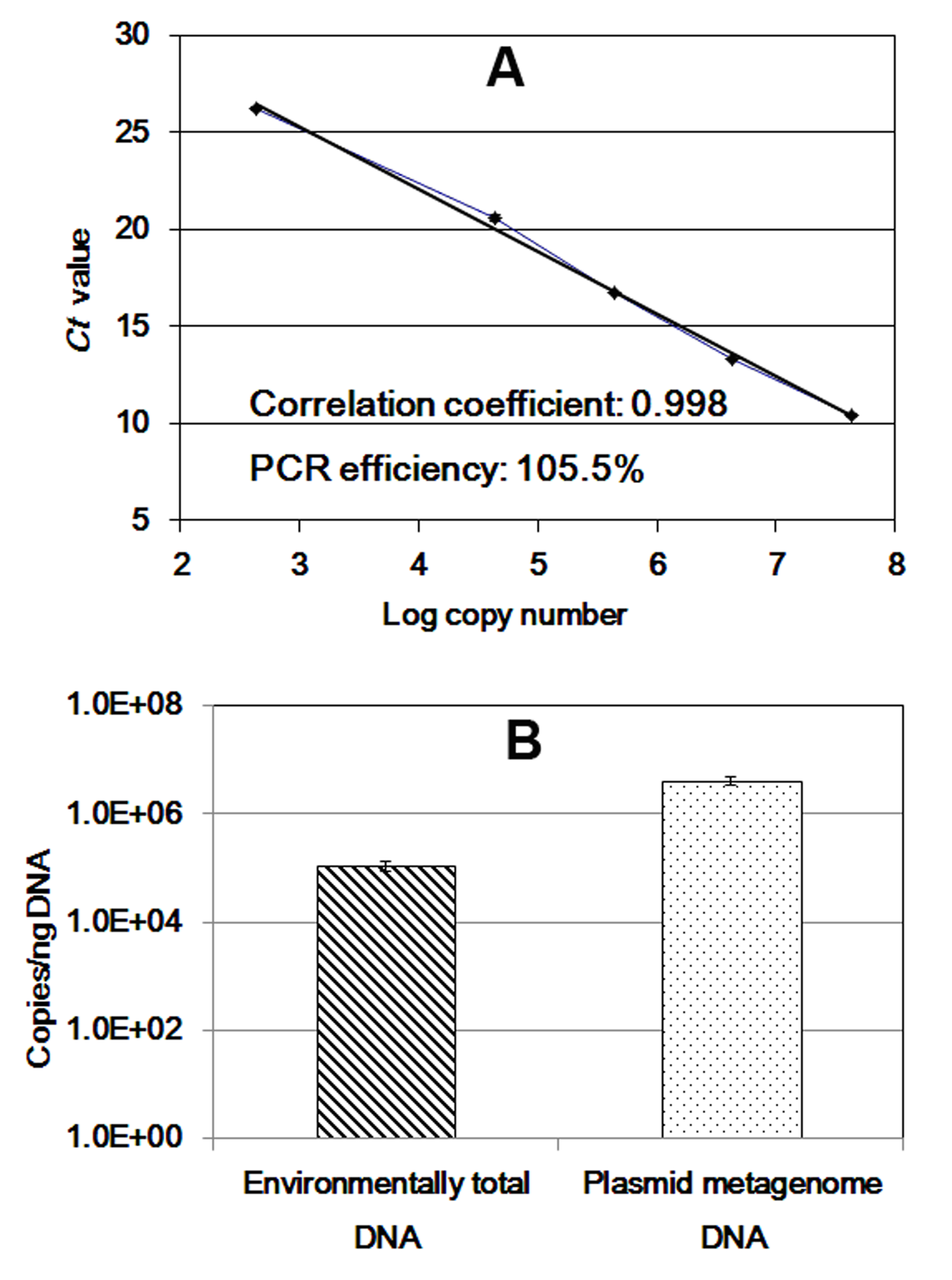

Supplement: Figure S1 — Calibration curves of quantitative real-time PCR for tetracycline resistance gene tetG generated with serial dilutions of plasmid vector carrying the target gene tetG (A) and comparison of tetG abundance in the environmentally total DNA and the concentrated plasmid metagenome isolated from activated sludge (B). (TIF) [file pone.0026041.s010.tif]

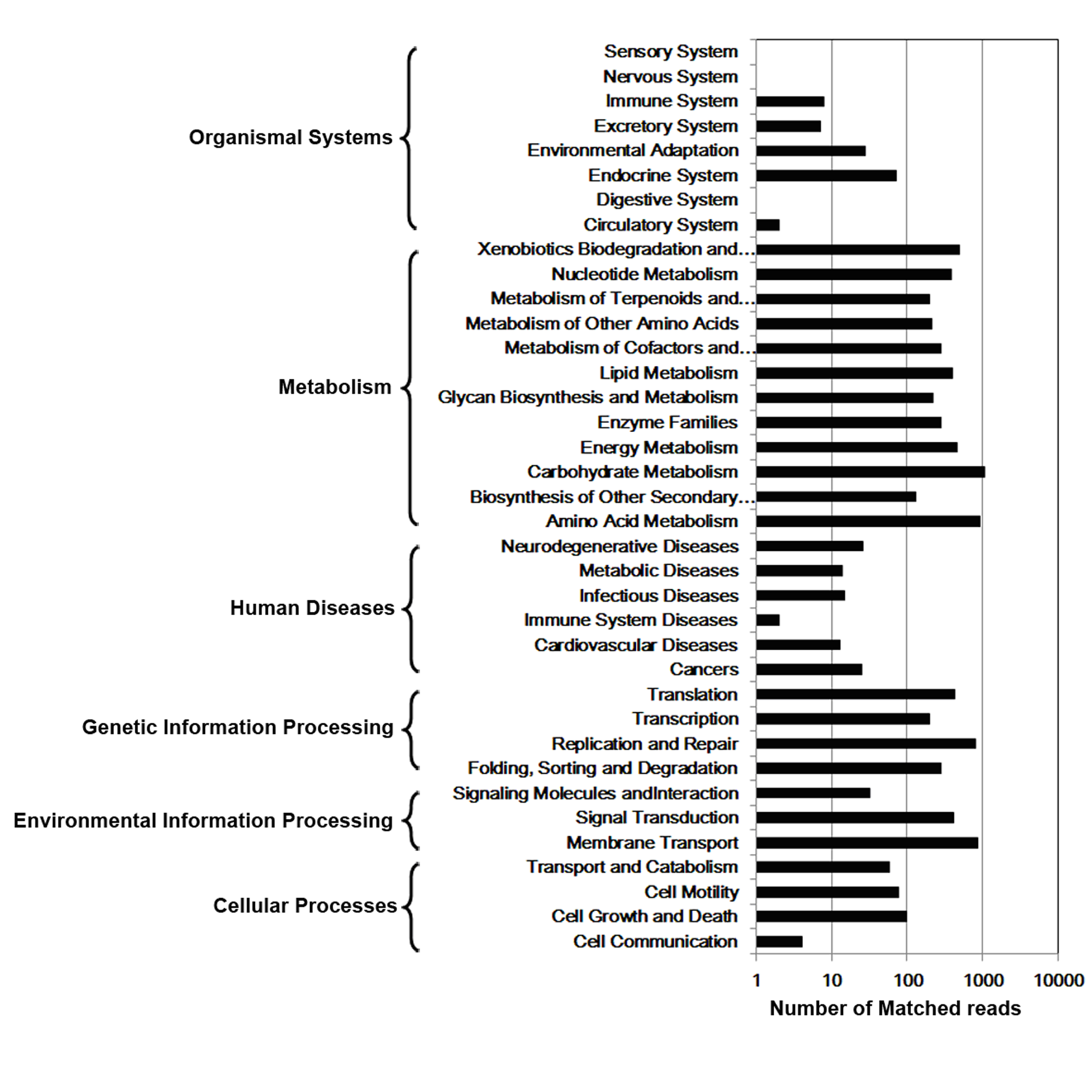

Supplement: Figure S2 — KEGG (Kyoto Encyclopedia of Genes and Genomes) pathway classification of plasmid metagenome of the activated sludge in Shatin STP (E-value cut off of 10−10). (TIF) [file pone.0026041.s011.tif]

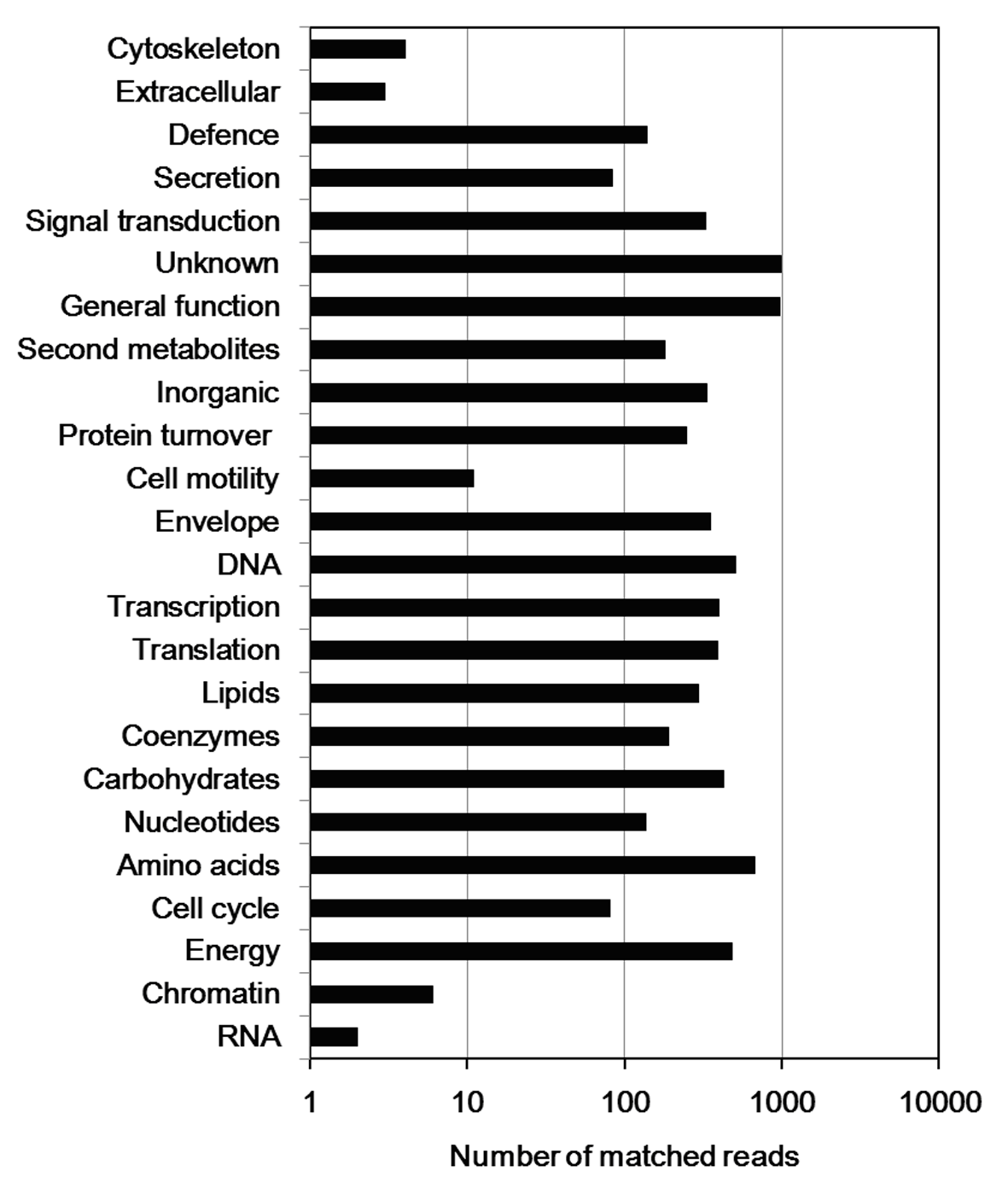

Supplement: Figure S3 — Functional classification of plasmid metagenome in the activated sludge of Shatin STP against evolutionary genealogy of genes: Non-supervised Orthologous Groups (eggNOG) databases (E-value cut-off of 10−10). (TIF) [file pone.0026041.s012.tif]

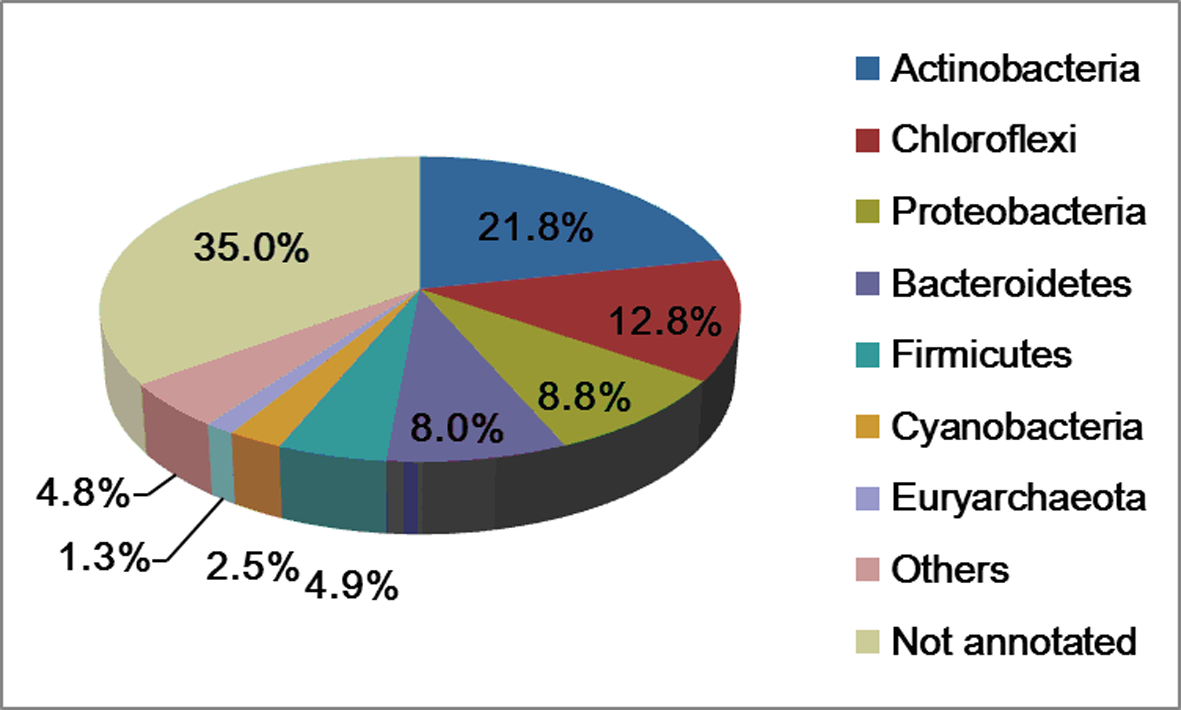

Supplement: Figure S4 — Species annotation of ORFs of plasmid metagenome in the activated sludge of Shatin STP against the non-redundant protein database at NCBI GenBank (E-value cut-off of 10−10). (TIF) [file pone.0026041.s013.tif]

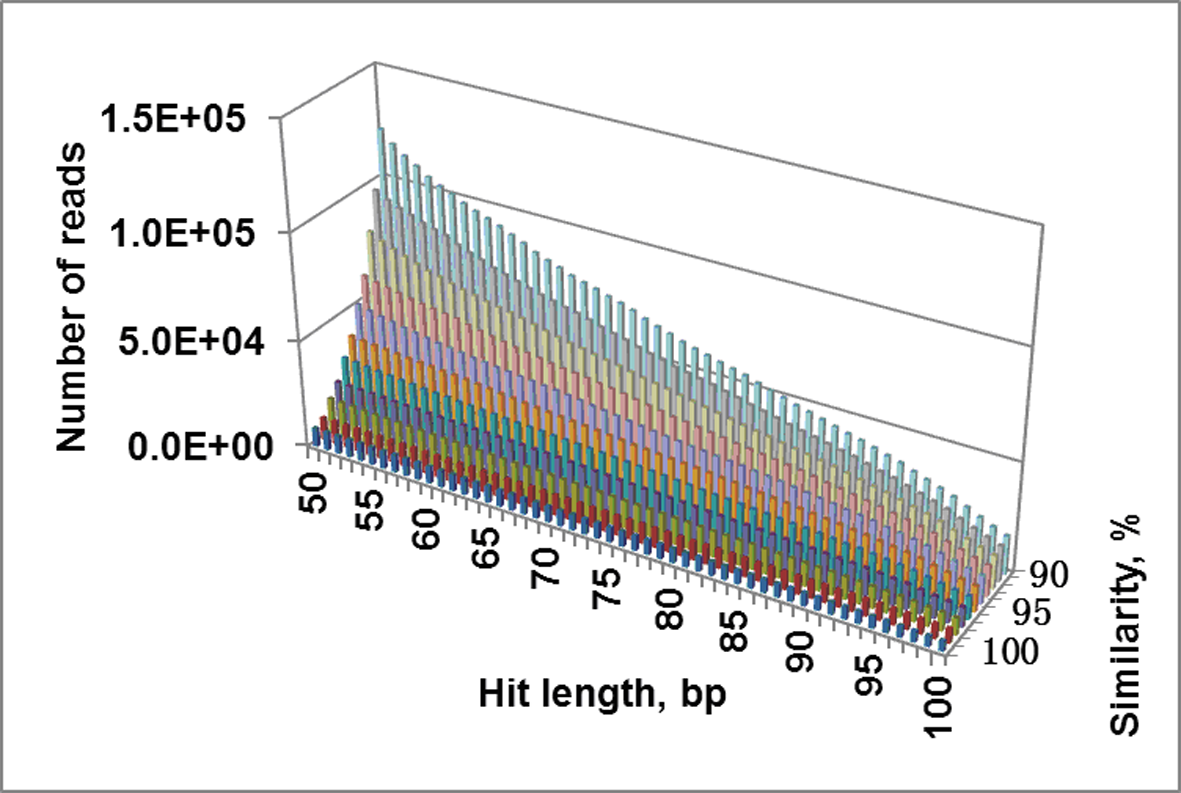

Supplement: Figure S5 — Number of matched high-throughput sequencing reads against NCBI plasmid genome database by using different cut-offs (hit length and sequence identity). The number of hits has significant negative correlation with both hit length and sequence identity. (TIF) [file pone.0026041.s014.tif]
